# Daily Rhythm of Mutualistic Pollinator Activity and Scent Emission in *Ficus septica*: Ecological Differentiation between Co-Occurring Pollinators and Potential Consequences for Chemical Communication and Facilitation of Host Speciation

**DOI:** 10.1371/journal.pone.0103581

**Published:** 2014-08-08

**Authors:** Lucie Conchou, Léa Cabioch, Lillian J. V. Rodriguez, Finn Kjellberg

**Affiliations:** 1 CEFE UMR 5175, CNRS - Université de Montpellier - Université Paul-Valéry Montpellier – EPHE, Montpellier, France; 2 Institute of Biology, University of the Philippines Diliman, Quezon City, Philippines; Institute of Vegetables and Flowers, Chinese Academy of Agricultural Science, China

## Abstract

The mutualistic interaction between *Ficus* and their pollinating agaonid wasps constitutes an extreme example of plant-insect co-diversification. Most *Ficus* species are locally associated with a single specific agaonid wasp species. Specificity is ensured by each fig species emitting a distinctive attractive scent. However, cases of widespread coexistence of two agaonid wasp species on the same *Ficus* species are documented. Here we document the coexistence of two agaonid wasp species in *Ficus septica*: one yellow-colored and one black-colored. Our results suggest that their coexistence is facilitated by divergent ecological traits. The black species is longer-lived (a few more hours) and is hence active until later in the afternoon. Some traits of the yellow species must compensate for this advantage for their coexistence to be stable. In addition, we show that the composition of the scent emitted by receptive figs changes between sunrise and noon. The two species may therefore be exposed to somewhat different ranges of receptive fig scent composition and may consequently diverge in the way they perceive and/or respond to scents. Whether such situations may lead to host plant speciation is an open question.

## Introduction

Plant-insect interactions are at the origin of huge diversification within the living world [1]. Plants need to attract pollinators and defend themselves against phytophagous insects, resulting in chemical diversification, and this diversity has led to radiations of whole insect groups. Despite the biological importance of this diversity, how plants and their associated insect communities diversify is largely unknown. For instance, what levels of ecological specialization will accelerate or impede the macroevolutionary diversification process is still largely unknown [2].

The mutualistic interaction between *Ficus* and agaonid wasps constitutes an extreme example of plant-insect co-diversification [3]. Agaonid wasps only develop in figs (the urn-shaped inflorescence of *Ficus*) of their host species (generally one host species, sometimes more [4]), and they are almost the sole pollinators of *Ficus* [5]. While many of the 800+ currently recognized *Ficus* species seem to be associated with a single pollinator species, some have two or more [6]. In some exceptional cases, co-pollinators of a *Ficus* species belong to different genera, a feature which should facilitate co-existence through strong ecological differentiation, as when diurnal *Elisabethiella* species coexist with nocturnal *Alfonsiella* species [7]. Much more often, the pollinators associated with a given *Ficus* species are sister species. The presence in different parts of the range of a *Ficus* species of different but closely related pollinator species could be an intermediate step in a process of allopatric speciation in which pollinator speciation could precede and maybe facilitate host speciation. In such situations, two or more species of pollinators may be observed to co-occur locally, but only in contact zones between their respective ranges. Examples include the three species of the *Wiebesia pumilae* complex associated with *Ficus pumila*, [8], and the two species associated to *Ficus sur* in West Africa. In the latter case, *Ceratosolen flabellatus* is a forest specialist, *C. silvestrianus* is a savanna specialist and both species coexist in forest-savanna mosaics [9].

In some situations, however, two or more sister species of pollinating wasps colonize the same trees and often even the same figs, over larger parts of the range of a *Ficus* species. In such situations, we may expect strong interspecific competition between the wasp species that should select for niche differentiation. For instance, co-occurring sister species could evolve different compromises between dispersal capacity (which in fig wasps is tightly linked to lifespan as these very short lived wasps disperse by drifting in the wind) and competitiveness (*e.g.* the ability to rapidly locate receptive figs, to enter them, and to oviposit faster than the other pollinator species).

There are five documented cases of relatively widespread co-occurrence of closely related pollinator species on a fig host, for which habitat differentiation has not been suggested as the mechanism allowing co-existence. The first example concerns *Ficus microcarpa* in Hainan [10] but no biological information or wasp description are provided and the evidence for widespread co-occurrence is preliminary. In Yunnan, however, one of the two sister species of agaonid wasps colonizing *F. microcarpa* does not carry pollen so that its larvae develop mainly in unfertilized fig ovules as opposed to mainly in fertilized ones in the case of pollinators [11], suggesting that, in this case, co-occurring sister species present highly divergent ecologies [12]. The second example concerns the co-occurrence of the sister species *Elisabethiella stuckenbergi* and *E. socotrensis* on *Ficus natalensis* in South Africa [4]. *E. stuckenbergi* has a shorter head than *E. socotrensis*, a characteristic that suggests different fig-entering capacities since head shape of agaonid wasps is strongly correlated with the shape of the entrance into the fig [13].

The three other examples involve differences in coloration among otherwise morphologically-similar wasp species. *Ficus tuerckheimii* is pollinated by two co-occurring species of *Pegoscapus* in both Mexico and Costa Rica [14]. *Pegoscapus carlosi* is uniformly black while *Pegoscapus mariae* is ventrally honey-colored and dorsally blackish [15]. Similarly, in Australia, *Ficus rubiginosa* is pollinated by four sister species constituting the *Pleistodontes imperialis* species complex [16]. The color varies from entirely dark testaceous (all specimens collected close to the city of Sydney, New South Wales), to dorsally testaceous and ventrally yellow (some specimens collected close to the city of Mareeba, Queensland), to nearly completely yellow (some specimens collected close to the city of Townsville, Queensland) [17]. The yellow-colored individuals correspond to one cytotype while the correspondence between color and cytotype is not yet ascertained for the other color morphs. Finally, *Ficus septica* is pollinated by a yellow and a black species that co-occur in south Taiwan [18], a situation we have also observed in populations from the Philippines. In both *F. septica* and *F. rubiginosa*, only the dark colored pollinators are present at the more temperate limits of their distributions.

In fig-pollinating wasps, light body color (qualified by authors as yellow, amber or honey) is almost always associated with large eyes and nocturnal flight, while dark color is always associated with smaller eyes and diurnal dispersal. In the three aforementioned cases however, both dark and light sister species have relatively small and similarly-sized eyes that suggest diurnal flight [15], [17], [19], [20]. In insects, melanisation or lack of melanisation may correlate with a diversity of adaptive traits [21]. Production of melanin is physiologically costly in insects: it can only be maintained if it confers a selective advantage [22]. For instance intra-population color variation in *Drosophila melanogaster* correlates strongly with resistance to dehydration, with light morphotypes being most sensitive [23]. The three cases reported above constitute the sole examples we have detected, in a systematic survey of pollinating fig wasp descriptions, of most probably diurnal light colored fig pollinating wasps. Therefore, we predict that the light color corresponds to an unusual life history strategy in diurnal pollinating fig wasps that is made possible because of selection for ecological niche separation between closely related species using the same resources. Given classical explanations of advantages associated with dark color, we surmise that dark pollinating wasps are better protected against diurnal stresses such as oxidative stress due to exposition to UV and ozone. We therefore predict that differences in body color might correlate with trade-offs between competitiveness and lifespan. Diurnal fig-pollinating wasps usually emerge from their natal fig early in the morning and survive only a few hours [24]–[26]. The black species, being better protected, would survive longer and have a more extended fig colonization time window than the yellow species. As a consequence it would be sufficiently long lived to reach somewhat more isolated receptive figs. Under the competitiveness-lifespan trade-off scenario, the yellow species would be more efficient than the black one at rapidly locating and entering receptive figs but would be shorter lived. A similar trade-off was found in the dark and pale males of the non-pollinating *Walkerella* sp. associated to *Ficus benjamina*, where males fight to access females [27]. Pale males were better fighters whereas dark males were more susceptible to disperse out of their natal figs in search for alternative mates. Dark males also tended to survive longer in laboratory conditions.

In *Ficus*, scent is the major cue used by pollinators to locate receptive figs and it facilitates the species-specificity of the interaction: each *Ficus* species produces a specific volatile blend that is only attractive to its specific pollinators [28]–[31]. However, variation of this volatile blend during the course of a day is expected as it has been reported for flowers as well as leaves of other plant species [32]–[34], and potential consequences of such variation on fig wasp behavior has not been investigated. One source of this variation originates in the plants being exposed, as the wasps, to a variety of stresses during daytime, involving oxidative stresses and thermal stresses. Volatile isoprenoids, including monoterpenes, protect plant tissues against these stresses [35], [36], and their emission varies during the day [37]. Monoterpenes are also perceived by insects and are involved in their attraction to plants (e.g. in fig wasps [38], [39]). We may therefore expect daily variation of the scents produced by receptive figs to result from responses to selection stemming from both daily patterns of pollinator activity and daily variation in abiotic stress intensity. On the contrary, leaves do not contribute to pollinator attraction [40] but they are exposed to the same abiotic stresses as figs. Daily leaf scent variation can therefore be used as a control to disentangle both functions in figs, and determine whether daily fig scent variation is the result of an adaption to increase pollinator attraction. Moreover, the presence of two pollinators instead of one is an exceptional situation for a fig species, and this may have selected for particular adaptations. It is therefore necessary to include a second type of control: a closely related *Ficus* species pollinated by a single wasp species.

Whatever the adaptive reasons are behind daily fig scent variation, we may expect adaptation of the pollinators to recognize the range of receptive fig scents to which they are regularly exposed. Two pollinator species presenting different daily activity patterns could be exposed to somewhat different ranges of attractive scent composition. If the hypothesis of more extended daily activity period of the black pollinators is upheld, and if scents emitted by receptive figs vary during the day, then we may predict some divergence over evolutionary times in the way the black and the yellow wasp species perceive and respond to volatile cues, a trait that could ultimately facilitate host plant speciation via assortative mating among plant genotypes if there is some heritable variation in receptive fig scent composition.

In this contribution, we document pollinator coexistence in *Ficus septica*. 1) We show that the yellow and black pollinator species associated with *Ficus septica* emerge from figs in the morning but differ in their lifespan, 2) we compare the daily rhythm of scent production by receptive figs with the rhythm of scent production by leaves and by figs and leaves of the closely-related *Ficus nota* and 3) we document that the composition of *Ficus septica* receptive fig scent varies during the course of a day. We discuss the potential consequences of these findings for the future evolution of this system, especially in terms of diversification processes.

## Materials and Methods

### Biology of the model system, study site and species

The fig is an urn-shaped inflorescence. Its inside is lined by uniovulate female flowers and male flowers. When the fig is receptive it emits a scent that attracts pollinating wasps. The wasps enter the fig, oviposit in some of the female flowers and pollinate. Female wasps that have colonized a fig and deposited their offspring in it are called foundresses. In monoecious *Ficus* species, seeds and galled flowers that each contains a single wasp larva develop side by side. Some weeks later, male wasp offspring emerge into the fig cavity and mate with the females still enclosed in their galls. The female wasps then emerge into the fig cavity, become loaded with pollen, and leave in search of a new, receptive fig. Finally, the fig ripens, becoming attractive to a large set of frugivorous animals. In dioecious *Ficus* species, functionally-male trees bear figs that produce wasps, pollen, but no seeds. Female trees bear figs that do not allow wasp oviposition and do not produce pollen, but do produce seeds. The adult lifespan of pollinating fig wasps is usually a few hours and is entirely devoted to searching for a receptive fig and subsequently ovipositing inside that fig. In *Ficus* in general, and in *Ficus septica* in particular, flowering is relatively synchronous within a given tree but asynchronous among trees resulting at the population level in the production of receptive figs and adult wasps throughout the year. The set of figs developing synchronously on a fig tree is called a crop.

The experimental work was carried out in the Diliman Campus of the University of the Philippines, in Quezon City, on the island of Luzon, Philippines (N 14°38′E 121°03′). Because the work was carried out in the campus of the University, no specific permit was required to conduct this study. Further, none of the studied species is protected or endangered. The campus is located in an urban zone and a large part of its area is made of more-or-less natural patches of vegetation. In the campus, native *Ficus* species grow wild in gardens, along roads and streams and in less intensively-tended places. Experimental work was carried out during the dry season, from January 14^th^ 2013 to April 12^th^ 2013. During this period, sunrise shifted from 6:30 a.m. to 5:45 a.m. and sunset from 5:45 p.m. to 6:15 p.m.

In Quezon City and more generally in the island of Luzon, *F. septica* is associated with two closely-related *Ceratosolen* species belonging to the *Ceratosolen bisulcatus* species complex (F. Kjellberg, L.J.V. Rodriguez unpublished observations, J.Y. Rasplus pers. com.): *Ceratosolen jucundus*, yellow-colored [19], and *Ceratosolen* sp., black-colored. Hereafter, they will be called “yellow pollinator” and “black pollinator” respectively.

In order to determine if the presence of two pollinators could have induced a shift in the period of the day when *Ficus septica* is pollinated and in the daily rhythm of fig scent production, some parallel observations were also done on a *Ficus* species associated to a single pollinator species. *Ficus nota* is taxonomically close to *F. septica* (both belong to subgenus *Sycomorus*, section *Sycocarpus*, subsection *Sycocarpus*) and is pollinated by the black-colored *Ceratosolen notus*, a close relative of the pollinators of *F. septica* [3]. Both species are found throughout the Philippines. A set of male trees of both species was surveyed every 2–3 days for the presence of figs and their developmental stage. Trees bearing figs close to receptivity or close to wasp emergence were visited daily.

### Traits associated with wasp lifespan and competitiveness

The aims of the following experiments were 1) to examine differences between black and yellow pollinators in traits associated with their daily activity patterns and 2) to test whether the daily patterns of yellow and black pollinator emergence and presence around receptive *Ficus septica* trees differ from those of the pollinator species associated to *Ficus nota*.

#### Wasp emergence patterns and lifespan of the black and yellow pollinators of *F. septica*

At 4–6 p.m., we collected male *F. septica* and *F. nota* figs from which wasps were to emerge on the following day. These figs were recognizable due to their swelling and their softness. Each fig was put into a separate plastic pot closed with plankton net. The figs were then stored outdoors under the shade of a tree so that physicochemical conditions were as close as possible to *in natura*. Wasp emergences from figs were recorded every hour on the following day, from 5 a.m. to sunset.

During the survey of wasp emergences, pots containing *F. septica* figs from which both black and yellow pollinators were emerging were put aside. The fig was removed from the pot in order to keep only the insects that had emerged during the preceding single hour. Dead pollinators were counted after 6 hours and every 3 hours thereafter until all the insects were dead. The survey was replicated on eight figs taken from five different mother trees on six different dates. Therefore, yellow and black species survival rates were first compared for each fig separately.

For every fig, all the insects were dead within 12 hours after their emergence. We therefore calculated for each fig the survival rate of emerging black and yellow pollinators at two time points: six and nine hours after emergence. To determine if one species had a longer lifespan than the other, we performed a binomial test for each time point: we ranked yellow and black species survival rate and transformed this ranking into a binomial variable B (B = 0 if yellow survival>black, B = 1 if black survival>yellow). Under the null hypothesis (yellow and black lifespan identical) we expected to observe B = 1 with a probability of 0.5 i.e. black survival rate to exceed yellow survival rate for just half of the figs. Our prediction was that black wasps would survive longer than yellow wasps (p(B = 1)>0.5).

#### Day round pattern of pollinating wasp presence on trees during their period of fig receptivity

In order to determine the day round presence on trees bearing receptive figs of the two pollinator species associated with *F. septica* and of the pollinator associated to *F. nota*, passive insect traps were hung in male trees bearing receptive figs. The insect traps were made of transparent A4 plastic sheets, rolled into a cylinder and coated with transparent odorless glue. Four such traps were suspended in branches close to receptive figs. The first insect traps were installed at 6 a.m., and were replaced every three hours until 6 a.m. on the following day. The experimental day was thus partitioned into eight time intervals. The exact timing of first trap setup, transition between fourth and fifth trap as well as last trap removal were adapted to match sunrise and sunset as close as possible. The experiment was repeated on six *F. septica* and three *F. nota* trees. After trap collection, the number of pollinators of each species captured during each three-hour time interval was counted.

Our prediction was that numbers of wasp trapped would decrease as the day progressed 1) because of limited lifespan of the wasps emerging in the morning and/or 2) because all the day's supply of receptive figs would no longer be attractive as they would have been pollinated in the morning. The following experiment was set up to discriminate between these two hypotheses.

#### Consequences of manipulating the period of accessibility of receptive *F. septica* figs on the abundance of black and yellow foundresses

Two branches bearing figs close to receptivity on each of three *F. septica* male trees were enclosed in plankton net bags for four to five days in order to let the figs become receptive without being pollinated. On one of the two branches per tree, the bag was removed at sunset the day before the experiment, at a time when pollinators were no longer active. Figs on this branch were thus accessible to pollinators for the whole experimental day. On the second branch the bag was removed at 0:30–1 p.m. on the day of the experiment, so that figs were accessible to pollinators only during the afternoon. All the figs were collected at sunset on the day of the experiment, and the number of black and yellow wasps that had penetrated each fig (number of foundresses) was determined. For each pollinator species, the mean number of foundresses per fig was compared between figs exposed the whole day and those exposed only in the afternoon, using Student's t-tests.

Our prediction was that because a lower proportion of the yellow pollinators would be alive and visiting figs in the afternoon than in the morning, figs exposed only during the afternoon would contain a higher proportion of black foundresses than fig already exposed to pollinators in the morning.

### Daily pattern of scent production by *F. septica* and *F. nota* at the time of fig receptivity

The aims of the following experiment were to establish 1) whether volatile organic compound (VOC) release by figs varied in quantity and/or in composition during the course of a day, 2) whether the pattern observed in figs is similar to the pattern observed in leaves or whether it is adjusted to the daily pattern of pollinator activity, and 3) whether *Ficus septica* figs display an unusual pattern (potential adaptive response to the presence of two pollinators using *Ficus nota* figs as a control).

#### Scent sampling design

We compared daytime variation in volatile emissions (sunrise and noon) between receptive male figs of *Ficus septica* (two pollinators) and *Ficus nota* (one pollinator) and between figs and leaves.

Pre-receptive figs were enclosed in plankton net bags in order to prevent pollination. Large numbers of pollinators flying around the tree was taken as a signal that many figs had become receptive. We then performed receptive fig scent and leaf scent extractions simultaneously, once at sunrise and then once at noon. This sampling protocol was repeated in five male individuals of each *Ficus* species.

Flowering phenology was somewhat asynchronous in both *Ficus* species, so that it was necessary to select the figs from which VOC emissions were collected in order to obtain a sample as homogenous as possible in developmental stage. To avoid any bias due to haphazard allocation of figs to sunrise and noon extractions, we randomized the selection process. Prior to each sunrise scent collection, 40 figs were chosen from the tree to be sampled and 20 of them were randomly assigned to the sunrise scent collection. The remaining 20 were used for the noon scent collection.

#### Scent extraction methods

Scent extraction was performed using the headspace technique [31]. The filters were designed to fit inside a chromatoprobe thermodesorption kit (see next section) and filled with 1.5 mg of Carbotrap 20–40 and 1.5 mg of Tenax 60–80. One microliter of a solution of internal standards (nonane and dodecane) in known concentrations was injected in each filter prior to extraction to allow later estimation of emission rates. Each sample was taken from either 20 receptive figs (selected as indicated above) or five leaves, cut off from branches and enclosed in polyethylene terephtalate bags. We standardized bag size to limit the variability in headspace volume and improve the repeatability of emission rate estimation. Scent was left to accumulate inside the bags for 30 minutes, and then the air was pulled out of the bag through the filter for five minutes with a flow rate of 160 mL/min. For each paired sample (one fig sample and one leaf sample extracted simultaneously), a control was made using an empty bag.

#### Identification and quantification of the volatile compounds

GC-MS analyses were carried out using a gas chromatograph CP-3800 (Varian Inc., Palo Alto, CA) equipped with an FID detector and coupled with a Saturn 2000 mass spectrometer (Varian). The samples were injected using a 1079 programmed temperature injector with a chromatoprobe kit (Varian), and was programmed as follow: 40°C hold for 0.5 min, and increased to 250°C at 200°C/min, hold for 3 min, and finally cooled down to 40°C with a fan. Chromatographic separation was performed using a fused silica capillary column (30 m×0.25 mm×0.25 µm Optima 5 Accent, Macherey-Nagel, Düren, Germany) with the following oven program: 40°C hold for 3 min, from 40°C to 100°C at 3.3°C/min, from 100 to 140°C at 2.9°C/min, from 140 to 180°C at 2.7°C/min, and finally upped to 250°C at 10°C/min and hold 8 min. The carrier gas was helium with a constant flow rate set close to 1.0 mL/min. The samples were injected in splitless mode. The energy for ionization by electron impact was 70 eV. The temperature of the transfer line, manifold and trap were respectively 250°C, 80°C and 170°C. The spectrometer was used in scan mode, from 38 to 300 m/z ratio.

All the volatile organic compounds (VOC) were tentatively identified by comparison with mass spectral libraries NIST98 MS and Adams 2007 [41], and retention indices found in Adams 2007 [41], online libraries (pherobase [42], NIST webbook [43]) and published data for (Z)- and (E)-DMNT [44]. Internal standards injected into each filter prior to scent extraction (0.08 µg nonane, 0.1 µg dodecane) allowed estimating the quantity (µg) of each identified compound contained in each sample.

#### Statistical analysis of scent profiles

Only VOCs that appeared in at least two different scent samples were retained to determine scent profiles. We checked that the presence/absence of the major VOCs was not affected by cutting off figs and leaves from branches. The only major VOC whose presence was due to cutting was (Z)-3-hexen-1-ol. Therefore, all the statistical analyses presented below were done after removing (Z)-3-hexen-1-ol from scent profiles. This had no qualitative effect on the results unless mentioned. From this VOC set, we calculated total emission rate and the relative composition of each scent profile. Total emission rates were the sum of emission rates of all VOCs detected in a given sample, calculated as µg/fig*hour for figs and as µg/cm^2^*hour for leaves. Relative scent composition was the relative contribution of each VOC to the scent profile, expressed as a percentage.

Emission rate variation among species and extraction hours were analyzed separately on figs and leaves, using Wilcoxon signed rank tests. Relative scent composition variation among species, organs, and extraction hours were analyzed with methods based on Bray-Curtis distances, implemented in the R package Vegan [45], [46]: Patterns of variation were visualized using non-metric multidimensional scaling (NMDS, [31]) and their significance tested with PERMANOVA [47]. NMDS is an ordination method which computes a locus for each sample within a space of given dimensionality so that the distances between samples on the final ordination are as close as possible to the original distances. The discrepancy between distances on the graph and actual distances is measured by the stress value, which varies from 0 (perfect correspondence) to 100% (no correspondence). According to the subset of samples to be included, we set the dimensionality to either 2 or 3 in order to always obtain stress values below 15%.

For the factors whose effect was detected to be significant by the PERMANOVA, we identified individual VOCs that contributed most to the overall difference by performing univariate Mann-Whitney tests between couples of sample categories. We tagged individual VOCs for which the p-value was lower than 0.05. We preferred this method to the dedicated test in the vegan package (simper function) because the latter is known to highlight variables presenting the highest intragroup variance rather than those that differ among groups [48]. Indeed, the simper function produced biologically meaningless results on our dataset.

## Results

### Traits associated with wasp lifespan and competitiveness

#### Wasp emergence patterns and lifespan of the black and yellow pollinators of *F. septica*

Most emergences from figs occurred in the morning in both *F. nota* and *F. septica*, independently of the pollinating wasp species. Indeed for 61% of 117 *F. septica* figs and for 57% of 89 *F. nota* figs, peak pollinator emergences occurred before 7 a.m. We did not detect any difference in timing of peak wasp emergence between *F. septica* figs hosting yellow, black or both pollinator species, and when both species emerged from the same fig they did so simultaneously. Since only 15 *F. septica* figs contained black pollinators (alone or together with yellow wasps), however, we could not exclude some slight difference in timing.

For all eight *Ficus septica* figs for which emerging pollinator lifespan was followed, all insects were dead within 12 hours after their emergence, so that survival was counted for each fig 6 hours and 9 hours after emergence. There was no single case of higher survival rate of yellow pollinators comparatively to black ones, resulting in globally significantly higher survival of black wasps 6 and 9 hours after emergence (Table 1).

**Table 1:** *Ficus septica* pollinating wasp lifespan survey: compared survival rates of yellow and black pollinators emerged from the same figs.

			survival rate (%)			
	number of wasps		6 hours after emergence		9 hours after emergence	
fig N°	yellow	black	yellow	black	yellow	black
1	28	150	28.57	78.66	0	5.33
2	106	24	56.60	75.00	0	4.16
3	136	39	84.55	100.00	0	30.76
4	29	28	48.27	89.28	0	0
5	31	125	77.41	81.60	0	0
6	77	36	58.44	88.88	14.28	38.88
7	214	57	77.10	98.24	5.14	75.43
8	203	25	46.79	60.00	0	12.00
binomial test p-value^1^			0.0039		0.016	

1 null hypothesis: if yellow and black pollinators have the same lifespan we expect that at any point in time the survival rate of the black species exceeds that of the yellow species in half of the replicates (p-values are for one-tailed tests, excluding ex-aequo).

#### Day round pattern of pollinating wasp presence on trees during their period of fig receptivity

**Figure 1:**
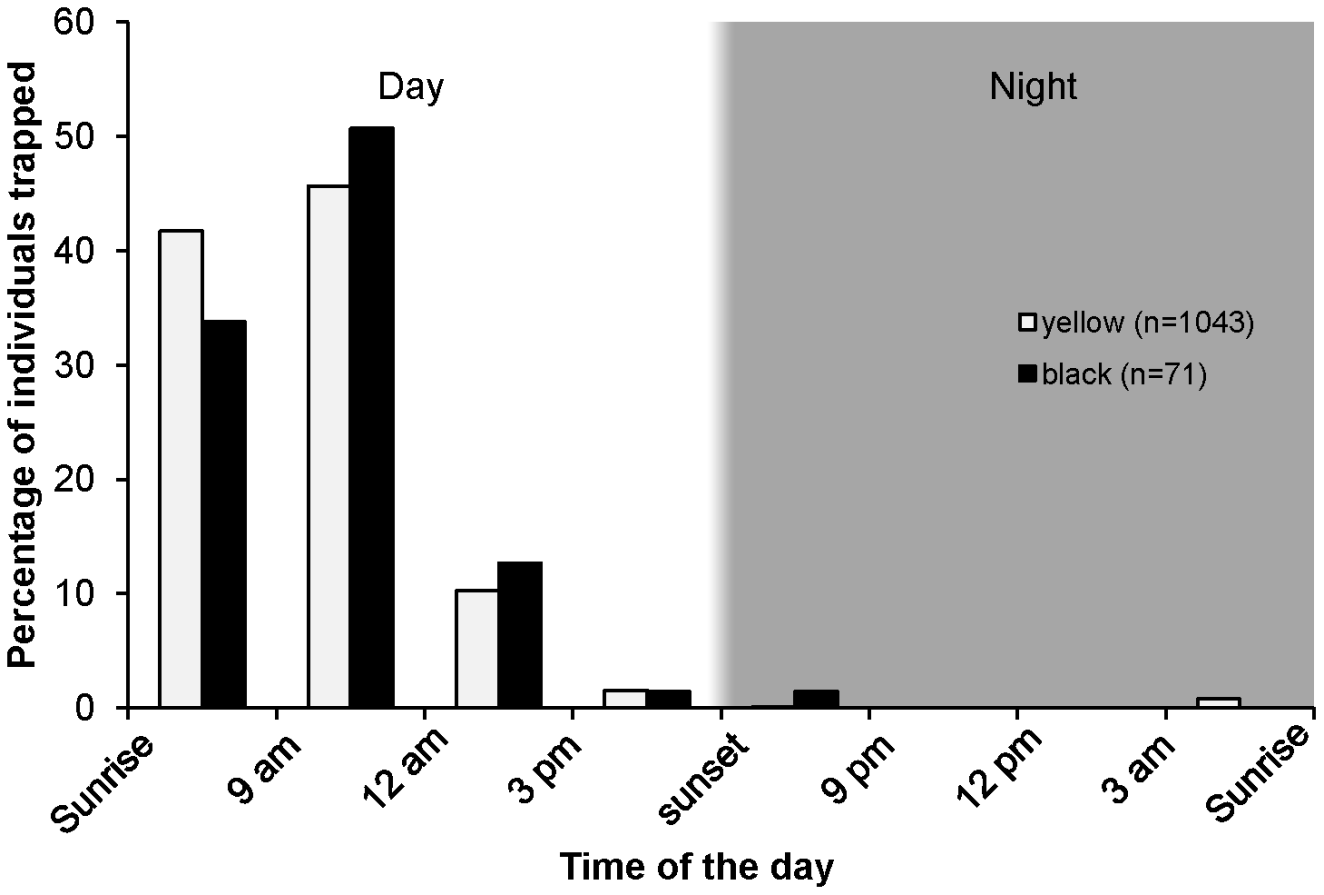
The daily pattern of *Ficus septica* pollinator activity around trees bearing receptive figs. Number of yellow and black pollinators trapped at different times of the day, expressed as percentage of the total number of individuals trapped over 24

Black *F. septica* pollinators were much less abundant than yellow ones during the field session (1043 yellow wasps caught versus 71 black ones), a pattern also observed when we were monitoring emergences. Both *F. septica* pollinators and *F. nota* pollinators were virtually always caught during daytime on sticky traps (Figure 1, Table S1). In *F. septica*, the presence patterns of black and yellow wasps were very similar (Figure 1): most individuals were caught in the morning (87% of yellow and 85% of black pollinators were caught between sunrise and 12 a.m., see Table S1 for detailed results), with detections decreasing during the afternoon and approaching zero during the night. Given the difference in lifespan between both species, we would have expected the relative frequency of black wasps around receptive trees to peak in the afternoon. However, in addition to wasp lifespan, the actual presence of attractive figs must also influence the daily patterns of wasp presence around receptive trees. Morning-pollinated figs could rapidly lose attractiveness, a feature that could explain the low numbers of black wasps trapped on the trees in the afternoon. The following experiment was set up to test that hypothesis.

#### Consequences of manipulating the timing of accessibility of *F. septica* figs on the abundance of black and yellow foundresses

**Figure 2:**
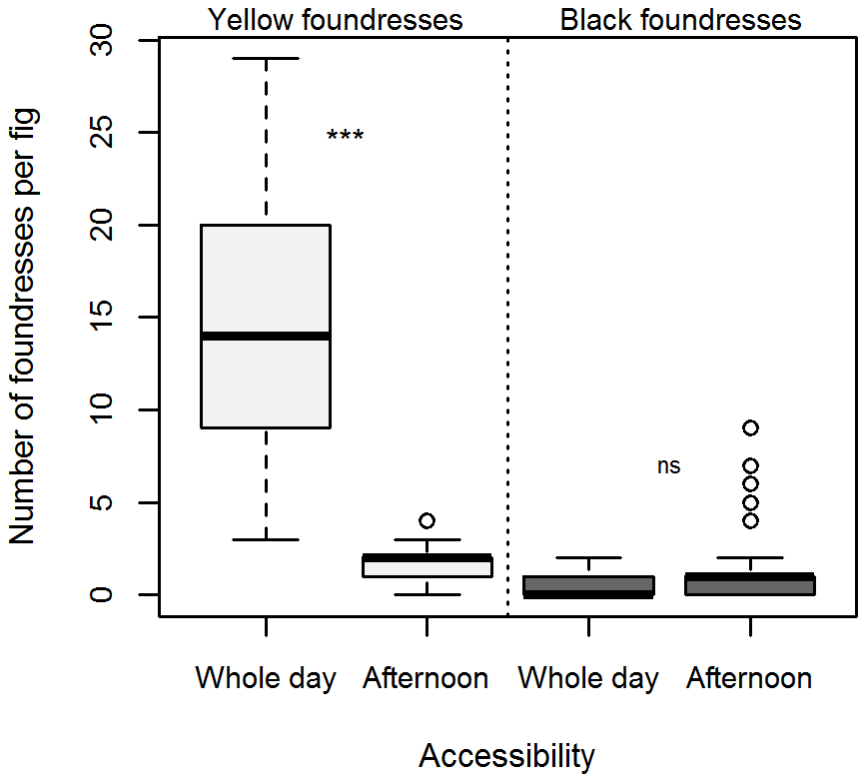
Colonization by pollinators of *Ficus septica* receptive figs whose accessibility has been manipulated. Number of yellow (light grey) and black (dark grey) foundresses found inside figs that have been left accessible to pollination for the whole day or in the afternoon only. Raw data provided as Table S2.

The figs that had been left accessible to wasps all day long contained many more yellow foundresses at the end of the experimental day than those that had been left accessible only during the afternoon (Wilcoxon rank sum test: W = 815, p<0.001, Figure 2). On the contrary, the number of black foundresses that had entered the figs did not differ according to their period of accessibility (W = 357.5, p = 0.35, Figure 2). The mean proportion of black wasps was significantly higher in figs left accessible only during the afternoon (whole day: 5+/−9% black wasps; afternoon: 34+/−28% black wasps; generalized linear model with quasibinomial distribution: t = −3.43, p = 0.0011). An interpretation of these results is that decreased pollinator densities in the afternoon in natural conditions could be due to a rapid loss of fig attractiveness once pollinated. We propose that when some figs remained attractive in the afternoon, black pollinators were more efficient at colonizing them probably because of their longer lifespan. Because wasp lifespan should be counted in hours, the longer longevity of black pollinators should enable them to colonize more distant host trees.

### Daily patterns of scent production by *F. septica* and *F. nota* at the time of fig receptivity

#### Variation in scent emission rates

**Figure 3:**
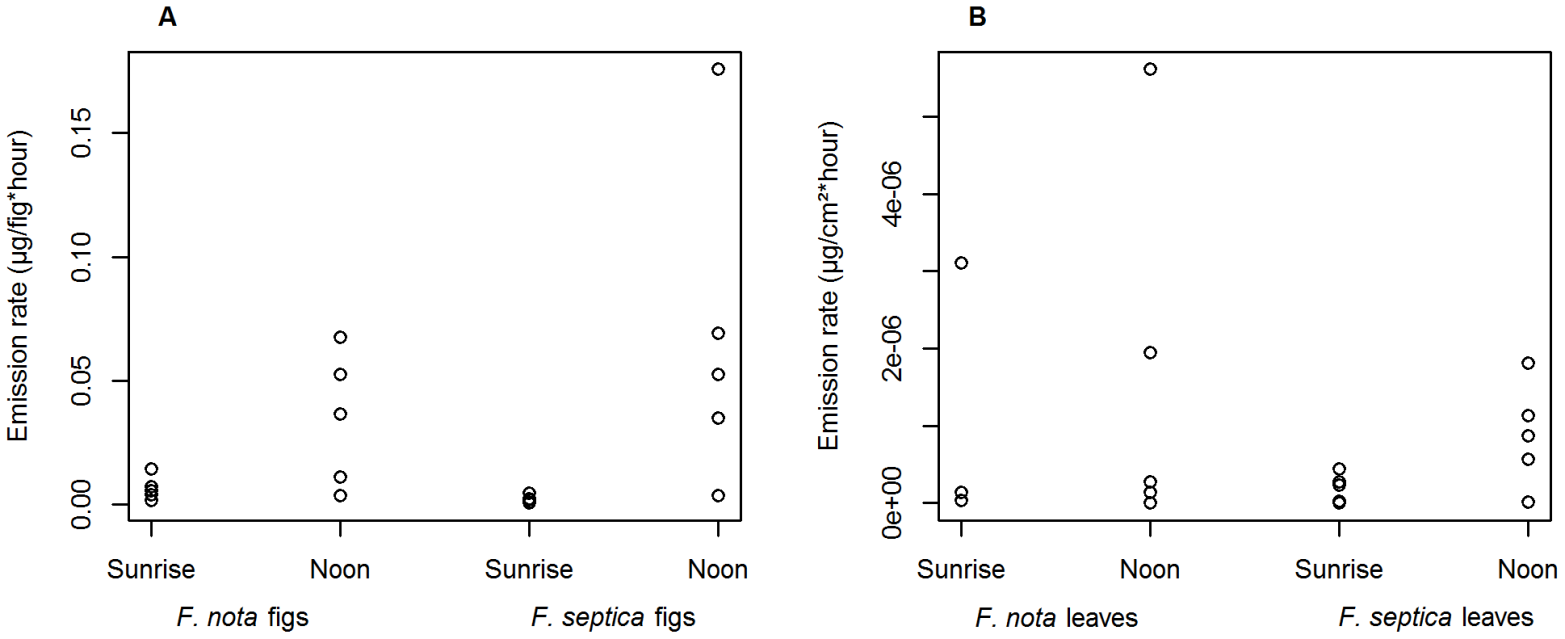
Daily variation of scent emission rates in *Ficus septica* and *Ficus nota*. Total scent emission rates from (A) figs (µg/fig*hour) and (B) leaves (µg/cm^2^*hour) of both species at sunrise and at noon.

Figs and leaves of the two species displayed the same pattern of variation: emission rates were significantly higher at noon than at sunrise (Wilcoxon signed rank test on both species pooled; figs: V = 8, p = 4.8*10^−5^, Figure 3.A.; leaves : Figure 3.B., V = 8, p = 0.049). Hence the general physiology of the trees and protection against abiotic stress is sufficient to explain the rhythm of fig VOC production. We have no evidence in favor of an emission rhythm that would reflect adaptation to pollinator activity rhythm or number of pollinator species.

#### Variation in relative scent composition

Seventy VOCs were detected in at least two samples and retained in the computation of scent profiles (Table S3, all statistical analyses exclude (Z)-3-hexen-1-ol, see material and methods). Forty-eight of them were tentatively identified. A further 16 of the non-identified ones were assigned to a biosynthetic category. Both *F. septica* and *F. nota* emitted mainly terpenoids (mean relative contribution varying from 48 to 94% depending on the species, organ and hour of extraction). Overall, figs emitted a larger number of different VOCs than leaves, and noon scents were comprised of a larger number of VOCs than morning scents (Table S3).

**Figure 4:**
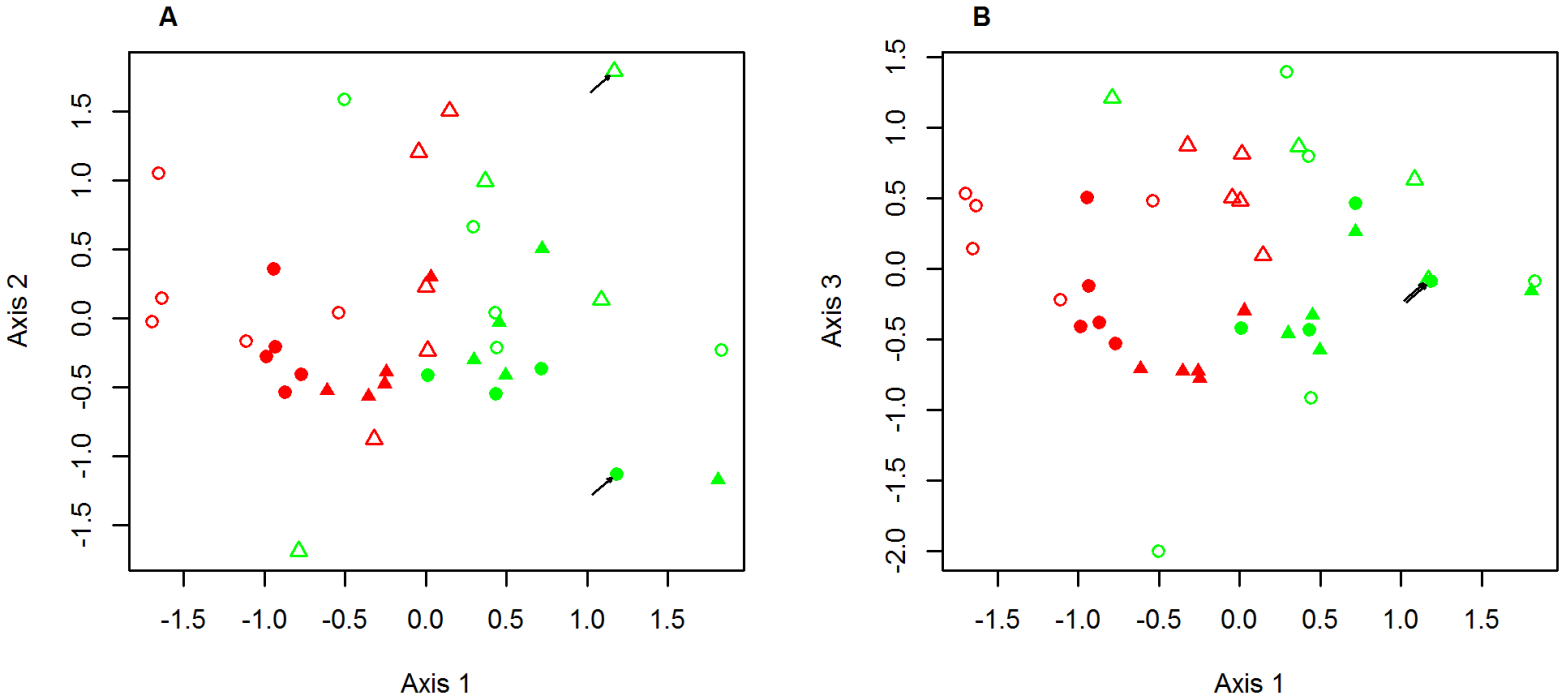
Patterns of variation of the composition of scents emitted by *Ficus septica* and *Ficus nota*. Three-dimensional NMDS ordination on the relative composition (% each VOC) of scents emitted by figs (red symbols) and leaves (green symbols) of *Ficus septica* (triangles) and *Ficus nota* (circles) at sunrise (open symbols) and at noon (closed symbols). (A) Axes 1 and 2, (B) Axes 1 and 3. Stress-value = 10%. Black arrows indicate places where two samples of the same category share the same locus.

Relative scent composition varied significantly according to species, organ type and time of extraction (global PERMANOVA, Table 2). The species*organ type and organ type*time of extraction interactions were also significant. On the 3 dimensional NMDS ordination, fig and leaf scents were separated along axis 1 (Figure 4). Morning and noon scents were separated on axes 2 (Figure 4.A) and 3 (Figure 4.B, Wilcoxon rank sum tests comparing position along the axes: W = 326 and p = 0.0004 for axis 2, W = 331 and p = 0.0002 for axis 3).

**Table 2:** PERMANOVA analysis on the relative composition of scents emitted by *Ficus septica* and *Ficus nota* figs and leaves at sunrise and noon.

Factor	Df	sum of squares	F-value	p-value
species	1	0.86	3.55	0.001
organ	1	1.56	6.45	1*10^−4^
hour	1	1.82	7.57	1*10^−4^
species*organ	1	0.67	2.78	0.007
species*hour	1	0.34	1.42	0.16
organ*hour	1	0.53	2.21	0.02
triple interaction	1	0.33	1.38	0.18
residuals	32	7.72		

In order to get better insights into sources of variation, we performed some further analyses separately on fig and leaf scents.

#### Separate analysis of daily variation in fig and leaf relative scent composition

**Figure 5:**
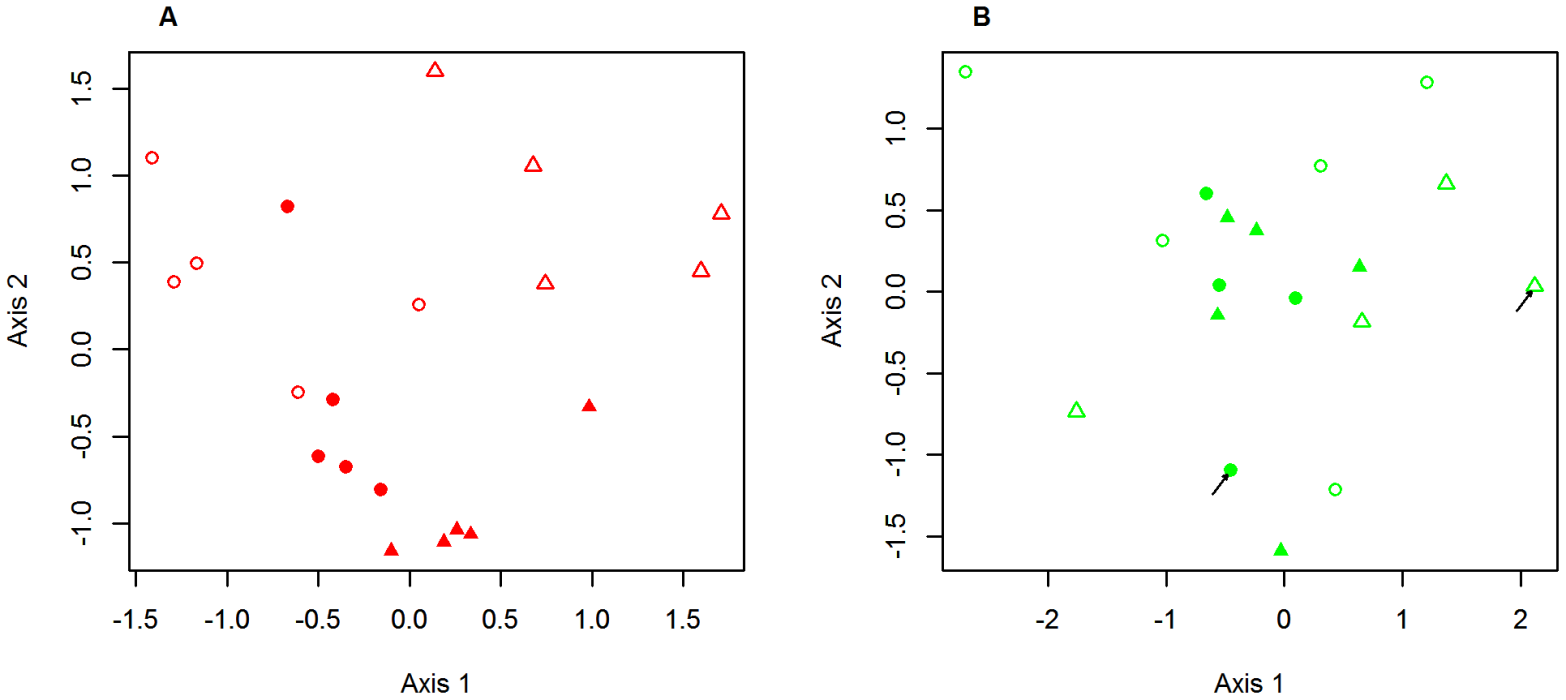
Variation in the composition of the scents emitted by figs (A) and leaves (B). Two-dimensional NMDS ordinations on relative scent composition (% each VOC) computed separately on (A) fig scents (stress-value = 14%) and (B) leaf scents (stress-value = 11%). Circles represent *Ficus nota* and triangles *Ficus septica* samples, open symbols are sunrise samples and closed symbols are noon samples. Black arrows indicate places where two samples of the same category share the same locus.

Sixty-seven VOCs were present in at least two fig samples. Receptive fig scent composition differed significantly between the two species and according to time of extraction (PERMANOVA, Table 3). The interaction term had no significant effect, suggesting that the two effects were orthogonal. Indeed, on the 2 dimensional NMDS ordination (Figure 5.A), the two species are separated along axis 1, and sunrise and noon samples along axis 2. Again, this suggests the absence of special features in *Ficus septica* scent production rhythm.

**Table 3:** Refined PERMANOVA analysis on the relative composition of scents emitted by figs of *Ficus septica* and of *Ficus nota* at sunrise and at noon.

Factor	Df	sum of squares	F-value	p-value
species	1	0.97	4.79	0.0005
hour	1	1.16	5.72	0.0002
species*hour	1	0.39	1.93	0.072
residuals	16	3.26	0.56	

Twenty VOCs were present in at least two leaf samples. Leaf scent composition varied significantly according to the hour of extraction, but the species effect and the interaction term were not significant (PERMANOVA, Table 4). Although we observed no segregation on the NMDS ordination (Figure 5.B.), sunrise samples were more dispersed than noon samples. Sunrise leaf scent composition was actually poorly consistent across samples, as only one VOC ((E)-caryophyllene) was detected in at least 4 out of 5 sunrise leaf samples in *F. nota*, and none in *F. septica*. In other words, we cannot define a clear mean profile for leaf scents in the morning. (Z)-3-hexen-1-ol was the only other compound consistently present in most morning leaf samples. When it was included in the analysis, sunrise and noon leaf scents were segregated along axis 1of the NMDS ordination (not shown). The lack of consistency in the composition of morning leaf scents may be due to low emission rates, as most compounds detected in these samples were at or near to the detection limits.

**Table 4:** Refined PERMANOVA analysis on the relative composition of scents emitted by leaves of *Ficus septica* and of *Ficus nota* at sunrise and at noon.

Factor	Df	sum of squares	F-value	p-value
species	1	0.47	1.72	0.10
hour	1	1.04	3.78	0.0015
species*hour	1	0.3	1.09	0.37
residuals	16	4.4	0.71	

#### Identification of the VOCs responsible for the difference between sunrise and noon scents

Regardless of species and organ type, the difference between sunrise and noon scent composition was mainly explained by a set of monoterpenes and both DMNT enantiomers (Table 5), whose relative proportion was higher at noon. The exact identity of the VOCs involved was different in figs of both species and in leaves, but (Z)- and (E)-β-ocimene were common to all. Consistent with this, there was a generalized increase in total monoterpenoid proportion at noon compared to sunrise scents, in figs of both species as well as in leaves (Table S3). In leaves, the relative proportion of (E)-DMNT was also higher at noon. This difference is due to a much stronger increase in the absolute quantity of several monoterpenes (and of (E)-DMNT in leaves) between sunrise and noon samples relative to other VOC categories (not shown). Consistent with the orthogonality of species and hour of extraction effects on fig scent composition, a set of sesquiterpenes were responsible for a large part of the difference in scent composition between figs of both species (Table S4).

**Table 5:** Main VOC responsible for the difference between sunrise and noon scent composition in figs of each species and in leaves.

	*F. nota* figs	*F. septica* figs	Leaves
*monoterpenes*			
sabinene	*	ns	ns
myrcene	*	*	ns
α-terpinene	*	ns	ns
1,8-cineole	*	ns	ns
(Z)-β-ocimene	*	**	*
(E)- β -ocimene	*	*	*
γ-terpinene	*	ns	ns
menthatriene	ns	*	ns
allo-ocimene	ns	**	*
*irregular terpenes*			
(Z)-DMNT	ns	ns	**
(E)-DMNT	ns	**	**
*phenylpropanoids/benzenoids*			
Aromatic 1	*	*	*
p-cymene	*	ns	ns
aromatic 2	*	ns	ns
p-cymenene	ns	*	ns
aromatic 5	ns	**	*
*unidentified*			
unidentified alcohol	ns	*	ns

P-values for the Mann-Whitney tests comparing the mean relative contribution of individual VOC to sunrise and to noon scent samples. Significance codes (values non corrected for multiple testing): ns = non-significant,

*<0.05,

**<0.01.

## Discussion

While it is rarely mentioned in the literature, field evidence shows that light colored fig pollinating wasps are generally nocturnal and dark colored fig pollinating wasps are diurnal [49]. To our knowledge, our results provide the first demonstration of the occurrence of a diurnal light colored fig pollinating wasp. This light colored fig pollinating wasp co-occurs on its host tree with a very closely related species that is dark colored. Studies using molecular markers have allowed detecting numerous cases in which several fig wasp species, generally qualified as cryptic, pollinate the same host [6]. We provide here one more example in which it is demonstrated that co-pollinators present divergent ecological traits, a feature which should facilitate co-existence. We suggest that most cases of several species of fig pollinating wasps co-occurring locally on a host will turn out to correspond either to contact zones between different species or to the co-occurrence of species presenting strongly divergent ecological traits. We show that the black pollinator species associated with *Ficus septi*ca is longer lived than the yellow species a feature which should enable it to drift further in the wind in search of receptive figs, and hence reach receptive figs located further away from the one they were born in. This should give some advantage to the black species comparatively to the yellow one. The two species coexist throughout the island of Luzon, and a similar situation is found in Taiwan with a yellow and a black species associated with *F. septica* [18]. This widespread coexistence can only be explained if some trait of the yellow species compensates for its shorter survival. We therefore predict that the yellow species is more efficient than the black species at rapidly locating, entering and/or ovipositing in receptive figs. If this competitiveness-lifespan trade-off hypothesis holds true, we would expect the black species to be more abundant in places where *Ficus septica* population density is low and the yellow species to be more abundant where population density is high. Hence, their coexistence would be facilitated by spatial heterogeneity of density of the resource they compete for. While *Ficus septica* fruits throughout the year, its fruiting frequency varies across seasons [50]. Therefore, we predict that the identity of the favoured species at a given location may also vary throughout the year.

We found published data and species descriptions suggesting the presence of the same pattern for the wasps associated with two other *Ficus* species: *F. rubiginosa* (subgenus *Urostigma* section *Malvanthera*) and wasp genus *Pleistodontes* in Australia, and *F. tuerkheimii* (subgenus *Urostigma* section *Americana*) and wasp genus *Pegoscapus* in America [14]–[17]. There are therefore potentially three independently evolved cases of diurnal light colored fig pollinating wasps pollinating a fig tree in competition with a very closely related black species. If diurnal behavior is confirmed, then it will be possible to test the prediction that in all three cases, the yellow species is shorter lived than the black one. Further investigations would then be needed to establish precisely what are the traits enabling yellow species persistence despite their shorter lifespan.

Other cases of *Ficus* species colonized by light colored and dark colored fig wasps are strikingly different. They include some species of section *Galoglychi*a pollinated by genera *Alfonsiella* and *Elisabethiella* [7]. But in those cases the *Alfonsiella* species are light colored and nocturnal and the *Elisabethiella* species are dark and diurnal. Similarly, *Ficus sycomorus* is visited by two agaonid wasps of genus *Ceratosolen. C. arabicus*, the pollinator, is light colored and nocturnal while the distantly related *C. galili* does not carry pollen and is dark colored and diurnal [26].

In order to establish whether the two wasp species pollinating *Ficus septica* encountered the same receptive fig scents, we analyzed daily variation of receptive fig scent composition in *F. septica* and in another fig species, *F. nota*, as a control. In both species the quantity of volatile compounds produced increased between morning and noon. Further the composition of the scent varied, notably the monoterpene content increased, i.e. compounds that are known to be detected by insects and among them fig wasps [38]–[39] and that are known to be used by insects to locate flowers. Hence, despite the central role of receptive fig scent in attracting fig wasps [28]–[31], this has not led to highly stereotyped receptive fig scents throughout the day. This new result is in agreement with previous studies which have evidenced strong variation within population among trees in receptive fig scent composition (one exception, *F. semicordata* uses a private channel to attract its pollinator [51]). Because of 1) the similar patterns of scent emission by the two *Ficus* species and 2) the similitude between figs and leaves in the daily pattern of scent variation, and 3) because monoterpenes are known to protect plant tissues against temperature and oxidative stresses [35], [36], we suggest that this variation is, at least in part, due to the production of volatile compounds protecting the organs against temperature and oxidative stresses. The biology of *Ficus sycomorus* suggests that the tree has limited control on the production of volatiles used by wasps to detect receptive figs. Indeed, *F. sycomorus* is pollinated at night by *Ceratosolen arabicus*, and in the daytime it is colonized by *C. galili*, a species that does not provide any pollination service. Despite this pattern of visitation, *F. sycomorus* receptive figs produced at noon the same main volatile compound and similar total quantities of volatile compounds as the closely related diurnally pollinated *F. sur* [31].

In this study, f we demonstrated that black and yellow pollinating wasps of *Ficus septica* have different lifespan and that receptive fig scent composition varies during the day. The two wasp species are therefore submitted to somewhat different ranges of receptive fig scents. We may therefore expect that they use a somewhat different range of chemical cues to locate receptive figs. If this is the case, then we may speculate on whether they respond differently to the within population variation of receptive fig scent among individual trees. Any such variation could lead to some assortative mating of the fig trees, thus structuring the gene flow within the *Ficus* species. Whether such processes are at work and whether they could ultimately lead to host speciation is an open question.

## Supporting Information

Table S1 **The daily rhythm of pollinator activity around trees bearing receptive figs: detailed results.**
(DOCX)

Table S2 **Tree by tree results of the experiment where the accessibility of receptive** ***Ficus septica*** **figs was manipulated.** Number of yellow and black foundresses found inside receptive figs that have been accessible to pollination either for the whole day or in the afternoon only.
(DOCX)

Table S3 **Mean relative composition of the scents emitted by figs and leaves of** ***Ficus septica*** **and** ***Ficus nota*** **at sunrise and at noon.** Mean relative contribution of each VOC to the scent of each sample category, expressed as a mean percentage +/− standard deviation.
(DOCX)

Table S4 **Main VOC responsible for additional differences of interest.** P-values for the Mann-Whitney tests comparing the mean relative contribution of individual VOC between categories indicated by column titles. Each comparison is between groups of 10 samples. Significance codes (values non corrected for multiple testing): ns = non significant, *<0.05, **<0.01, ***<0.001.
(DOCX)

## References

[1] Stone GN, Lohse K, Nicholls JA, Fuentes-Utrilla P, Sinclair F, et al (2012) Reconstructing community assembly in time and space reveals enemy escape in a Western Palearctic insect community. Current Biology 22: 532–537. 22405865 10.1016/j.cub.2012.01.059

[2] Forister ML, Dyer LA, Singer MS, Stireman JO, Lill JT (2012) Revisiting the evolution of ecological specialization, with emphasis on insect–plant interactions. Ecology 93: 981–991. 22764485 10.1890/11-0650.1

[3] Cruaud A, Ronsted N, Chantarasuwan B, Chou LS, Clement WL, et al (2012) An extreme case of plant-insect co-diversification: figs and fig-pollinating wasps. Syst Biol 61: 1029–1047. 22848088 10.1093/sysbio/sys068 PMC3478567

[4] Cornille A, Underhill JG, Cruaud A, Hossaert-McKey M, Johnson SD, et al (2012) Floral volatiles, pollinator sharing and diversification in the fig-wasp mutualism: insights from *Ficus natalensis* and its two wasp pollinators (South Africa). Proc R Soc Lond B Biol Sci 279: 1731–1739. 10.1098/rspb.2011.1972 PMC3297447 22130605

[5] Jousselin E, Rasplus JY, Kjellberg F (2001) Shift to mutualism in a parasitic lineage of the fig/fig wasp interaction. Oikos 94: 287–294.

[6] Herre EA, Jandér KC, Machado CA (2008) Evolutionary ecology of figs and their associates: recent progress and outstanding puzzles. Annu Rev Ecol Evol Syst 39: 439–458.

[7] Compton SG, Grehan K, van Noort S (2009) A fig crop pollinated by three or more species of agaonid fig wasps. Afr Entomol 17: 215–222.

[8] Chen Y, Compton SG, Liu M, Chen XY (2012) Fig trees at the northern limit of their range: the distributions of cryptic pollinators indicate multiple glacial refugia. Mol Ecol 21: 1687–1701. 22335780 10.1111/j.1365-294X.2012.05491.x

[9] Kerdelhué C, Le Clainche I, Rasplus JY (1999) Molecular phylogeny of the *Ceratosolen* species pollinating *Ficus* of the subgenus *Sycomorus* sensu stricto: biogeographical history, and origins of the species-specificity breakdown cases. Mol Phylogenet and Evol 11: 401–414. 10.1006/mpev.1998.0590 10196081

[10] Sun XJ, Xiao JH, Cook JM, Feng G, Huang DW (2011) Comparisons of host mitochondrial, nuclear and endosymbiont bacterial genes reveal cryptic fig wasp species and the effects of *Wolbachia* on host mtDNA evolution and diversity. BMC Evol Biol 11: 86. 21457543 10.1186/1471-2148-11-86 PMC3083349

[11] Jousselin E, Hossaert-McKey M, Herre EA, Kjellberg F (2003) Why do fig wasps actively pollinate monoecious figs? Plant Animal Interactions 134: 381–387. 10.1007/s00442-002-1116-0 12647146

[12] Martinson EO, Jandér KC, Peng YQ, Chen HH, Machado CA, et al (2014) Relative investment in egg load and poison sac in fig wasps: Implications for physiological mechanisms underlying seed and wasp production in figs. Acta Oecol 57: 58–66 http://dx.doi.org/10.1016/j.actao.2013.07.009.

[13] Van Noort S, Compton SG (1996) Convergent evolution of agaonine and sycoecine (Agaonidae, Chalcidoidea) head shape in response to the constraints of host fig morphology. J Biogeogr 23: 415–424.

[14] Ramirez WB (1970) Host specificity of fig wasps (Agaonidae). Evolution 24: 680–691. 28564937 10.1111/j.1558-5646.1970.tb01804.x

[15] Ramirez WB (1970) Taxonomic and biological studies of neotropical fig wasps (Hymenoptera: Agaonidae). The University of Kansas Science Bulletin 49: 1–44.

[16] Haine ER, Martin J, Cook JM (2006) Deep mtDNA divergences indicate cryptic species in a fig pollinating wasp. BMC Evol Biol 6: 83. 17040562 10.1186/1471-2148-6-83 PMC1626083

[17] Lopez-Vaamonde C, Dixon DJ, Cook JM, Rasplus JY (2002) Revision of the Australian species of *Pleistodontes* (Hymenoptera: Agaonidae) fig-pollinating wasps and their host-plant associations. Zool J Linn Soc 136: 637–683.

[18] Lin RC, Yeung CKL, Fong JJ, Tzeng HY, Li SH (2011) The lack of pollinator specificity in a dioecious fig tree: sympatric fig-pollinating wasps of *Ficus septica* in Southern Taiwan. Biotropica 43: 200–207.

[19] Grandi G (1927) Hyménoptères sycophiles récoltés aux iles Philippines par C.F. Baker, I. Agaonini. The Philipp J S 33: 309–329.

[20] Grandi G (1928) Revisione critica degli Aganidi descritti da Gustavo Mayr e catalogo ragioanto delle species fino ad oggi descritte di tutto il mondo. Bollettino del Laboratorio di Entomologia del R.Istituto Superiore Agrario di Bologna 1: 107–235.

[21] True JR (2003) Insect melanism: the molecules matter. Trends Ecol Evol 18: 640–647.

[22] Roff DA, Fairbairn DJ (2013) The costs of being dark: the genetic basis of melanism and its association with fitness-related traits in the sand cricket. J Evol Biol 26: 1406–1416. 23675858 10.1111/jeb.12150

[23] Parkash R, Rajpurohita S, Ramniwas S (2009) Impact of darker, intermediate and lighter phenotypes of body melanization on desiccation resistance in *Drosophila melanogaster* . J Insect Sci 9: 49. 10.1673/031.009.4901 PMC3011941 20050769

[24] Kjellberg F, Doumesche B, Bronstein J (1988) Longevity of a fig wasp (*Blastophaga psenes*). Ecology 91: 117–122.

[25] Ghara M, Borges R (2010) Comparative life-history traits in a fig wasp community: implications for community structure. Ecol Entomol 35: 138–148.

[26] Warren M, Robertson MP, Greef JM (2010) A comparative approach to understanding factors limiting abundance patterns and distribution in fig tree-fig wasp mutualism. Ecography 33: 148–158.

[27] Wang ZJ, Peng YQ, Compton SG, Yang DR (2010) Reproductive strategies of two forms of flightless males in a non-pollinating fig wasp under partial local mate competition. Ecol Entomol 35: 691–697.

[28] Wang G, Compton SG, Chen J (2013) The mechanism of pollinator specificity between two sympatric fig varieties: a combination of olfactory signals and contact cues. Ann Bot 111: 173–181. 23179860 10.1093/aob/mcs250 PMC3555521

[29] Proffit M, Chen C, Soler C, Bessière JM, Schatz B, et al (2009) Can chemical signals responsible for mutualistic partner encounter promote the specific exploitation of nursery pollination mutualisms? The case of figs and fig wasps. Entomol Exp Appl 131: 46–57 (doi:10.1111/j.1570-7458.2009.00823.x)

[30] Grison L, Edwards AA, Hossaert-McKey M (1999) Interspecies variation in floral fragrances emitted by tropical *Ficus* species. Phytochemistry 52: 1293–1299.

[31] Proffit M, Johnson SD (2009) Specificity of the signal emitted by figs to attract their pollinating wasps: comparison of volatile organic compounds emitted by receptive syconia of *Ficus sur* and *F. sycomorus* in Southern Africa. S Afr J Bot 75: 771–777.

[32] Loivamäki M, Louis S, Cinege G, Zimmer I, Fischbach R, et al (2007) Circadian rythms of isoprene biosynthesis in grey poplar leaves. Plant Physiol 143: 540–551. 17122071 10.1104/pp.106.092759 PMC1761966

[33] Helsper JPFG, Davies JA, Bouwmeester HJ, Krol AF, van Kampen MH (1998) Circadian rhythmicity in emission of volatile compounds by flowers of *Rosa hybrida* L. cv. Honesty. Planta 207: 88–95.

[34] Loreto F, Schnitzler JP (2010) Abiotic stresses and induced BVOCs. Trends Plant Sci 15: 154–166. 20133178 10.1016/j.tplants.2009.12.006

[35] Loreto F, Pinelli P, Manes F, Kollist H (2004) Impact of ozone on monoterpene emissions and evidence for an isoprene-like antioxidant action of monoterpenes emitted by *Quercus ilex* leaves. Tree Physiol 24: 361–367. 14757575 10.1093/treephys/24.4.361

[36] Copolovici LO, Filella I, Llusià J, Niinemets U, Peñuelas J (2005) The capacity for thermal protection of photosynthetic electron transport varies for different monoterpenes in *Quercus ilex* . Plant Physiol 139: 485–496. 16126854 10.1104/pp.105.065995 PMC1203397

[37] Staudt M, Bertin N, Hansen U, Seufert G, Ciccioli P, et al (1997) Seasonal and diurnal patterns of monoterpene emissions from *Pinus pinea* (L.) under field conditions. Atmos Environ 31: 145–156.

[38] Soler CCL, Proffit M, Bessière JM, Hossaert-McKey M, Schatz B (2012) Evidence for intersexual chemical mimicry in a dioecious plant. Ecol Lett 15: 978–985. 22762353 10.1111/j.1461-0248.2012.01818.x

[39] Chen C, Song Q (2008) Responses of the pollinating wasp *Ceratosolen solmsi marchali* to odor variation between two floral stages of *Ficus hispida* . J Chem Ecol 34: 1536–1544. 19015920 10.1007/s10886-008-9558-4

[40] Song Q, Yang D, Zhang G, Yang C (2001) Volatiles from *Ficus hispida* and their attractiveness to fig wasps. J Chem Ecol 27: 1929–1942. 11710602 10.1023/a:1012226400586

[41] Adams RP (2007) Identification of Essential Oil Components by Gas Chromatography/Mass Spectrometry, Fourth Ed. Allured Publishing Corp, Carol Stream, Illinois, USA.

[42] El-Sayed AM (2012) The Pherobase: Database of Pheromones and Semiochemicals. Available: http://www.pherobase.com. Accessed 2013 May–Jun.

[43] Linstrom PJ, Mallard WG, editors. NIST Chemistry WebBook, NIST Standard Reference Database Number 69, National Institute of Standards and Technology, Gaithersburg MD, 20899. Available: http://webbook.nist.gov. Accessed 2013 May–Jun.

[44] Delle-Vedove R, Juillet N, Bessière JM, Grison C, Barthes N, et al (2011) Colour-scent associations in a tropical orchid: Three colours but two odours. Phytochemistry 72: 735–742. 21377705 10.1016/j.phytochem.2011.02.005

[45] Oksanen J, Blanchet G, Kindt R, Legendre P, Minchin PR, et al. (2013) vegan: Community Ecology Package. R package version 2.0-7. Available: http://CRAN.R-project.org/package=vegan. Accessed June 2013.

[46] R Core Team (2013) R: A language and environment for statistical computing. R Foundation for Statistical Computing, Vienna, Austria. Available: http://www.R-project.org/.

[47] Anderson MJ (2001) A new method for non-parametric multivariate analysis of variance. Austral Ecol 26: 32–46.

[48] Warton DI, Wright TW, Wang Y (2012) Distance-based multivariate analyses confound location and dispersion effects. Methods Ecol Evol 3: 89–101.

[49] Harrison RH, Rasplus JY (2006) Dispersal of fig pollinators in asian tropical rain forests. J Trop Ecol 22: 631–639.

[50] Bain A, Chou LS, Tzeng HY, Ho YC, Chiang YP (2014) Plasticity and diversity of the phenology of dioecious *Ficus* species in Taiwan. Acta Oecol 57: 124–134.

[51] Chen C, Song Q, Proffit M, Bessière JM, Li Z, et al (2009) Private channel : a single unusual compound assures specific pollinator attraction in *Ficus semicordata* . Funct Ecol 23: 941–950.

