# Supplementary material for: Daily Rhythm of Mutualistic Pollinator Activity and Scent Emission in Ficus septica: Ecological Differentiation between Co-Occurring Pollinators and Potential Consequences for Chemical Communication and Facilitation of Host Speciation
Source: PLoS One. 2014 Aug 8;9(8):e103581. doi: 10.1371/journal.pone.0103581 (PMC4126690; doi:10.1371/journal.pone.0103581)
Supplement: Table S1 — The daily rhythm of pollinator activity around trees bearing receptive figs: detailed results. (DOCX) [file pone.0103581.s001.docx]

Table S1: The daily rhythm of pollinator activity around trees bearing receptive figs: detailed results.

- Number of black and yellow pollinators trapped on *Ficus septica* trees according to the time of the day. The experiment was conducted on 6 different trees at 6 different dates. Total number of pollinators trapped varied greatly from one tree to the next but the trends were similar.

| Pollinator  species |  | Number of pollinators caught on sticky traps | | | | | | | | |
| --- | --- | --- | --- | --- | --- | --- | --- | --- | --- | --- |
|  | Tree N° | sunrise-9 am | 9-12 am | 12 am-3 pm | 3 pm-sunset | sunset-9 pm | 9-12 pm | 12 pm-3 am | 3 am-sunrise | **total** |
| yellow | 1 | 17 | 39 | 8 | 1 | 1 | 0 | 0 | 8 | **74** |
|  | 2 | 3 | 2 | 0 | 0 | 0 | 0 | 0 | 0 | **5** |
|  | 3 | 145 | 219 | 40 | 6 | 0 | 0 | 0 | 0 | **410** |
|  | 4 | 48 | 39 | 3 | 0 | 0 | | | | **90** |
|  | 5 | 25 | 54 | 34 | 8 | 0 | | | | **121** |
|  | 6 | 197 | 123 | 22 | 1 | 0 | | | | **343** |
|  | **sum** | **435** | **476** | **107** | **16** | **1** | **0** | **0** | **8** | **1043** |
|  | **% of total** | **42** | **46** | **10** | **2** | **0** | **0** | **0** | **1** | **100** |
| black | 1 | 0 | 1 | 0 | 0 | 1 | 0 | 0 | 0 | **2** |
|  | 2 | 0 | 0 | 0 | 1 | 0 | 0 | 0 | 0 | **1** |
|  | 3 | 0 | 1 | 1 | 0 | 0 | 0 | 0 | 0 | **2** |
|  | 4 | 2 | 3 | 0 | 0 | 0 | | | | **5** |
|  | 5 | 3 | 9 | 7 | 0 | 0 | | | | **19** |
|  | 6 | 19 | 22 | 1 | 0 | 0 | | | | **42** |
|  | **sum** | **24** | **36** | **9** | **1** | **1** | **0** | **0** | **0** | **71** |
|  | **% of total** | **34** | **51** | **13** | **1** | **1** | **0** | **0** | **0** | **100** |

- Number of pollinators trapped around *Ficus nota* trees according to the time of the day. The experiment was conducted on 3 different trees at 3 different dates.

|  | Number of pollinators caught | | | | | | | | |
| --- | --- | --- | --- | --- | --- | --- | --- | --- | --- |
| Tree N° | sunrise-9 am | 9-12 am | 12 am-3 pm | 3 pm-sunset | sunset-9 pm | 9-12 pm | 12 pm-3 am | 3 am-sunrise | total |
| 1 | 54 | 105 | 100 | 23 | 1 | 1 | 0 | 6 | 290 |
| 2 | 66 | 49 | 43 | 35 | 1 | 0 | 0 | 7 | 201 |
| 3 | 105 | 37 | 25 | 24 | 1 | | | | 192 |
| **sum** | **225** | **191** | **168** | **82** | **3** | **1** | **0** | **13** | **683** |
| **% of total** | **33** | **28** | **25** | **12** | **0** | **0** | **0** | **2** | **100** |
