# Supplementary material for: Daily Rhythm of Mutualistic Pollinator Activity and Scent Emission in Ficus septica: Ecological Differentiation between Co-Occurring Pollinators and Potential Consequences for Chemical Communication and Facilitation of Host Speciation
Source: PLoS One. 2014 Aug 8;9(8):e103581. doi: 10.1371/journal.pone.0103581 (PMC4126690; doi:10.1371/journal.pone.0103581)
Supplement: Table S2 — Tree by tree results of the experiment where the accessibility of receptive Ficus septica figs was manipulated. Number of yellow and black foundresses found inside receptive figs that have been accessible to pollination either for the whole day or in the afternoon only. (DOCX) [file pone.0103581.s002.docx]

Table S2: Tree by tree results of the experiment where the accessibility of receptive *Ficus septica* figs was manipulated. Number of yellow and black foundresses found inside receptive figs that have been accessible to pollination either for the whole day or in the afternoon only.

|  |  | Tree 1 | | Tree 2 | | Tree 3 | |
| --- | --- | --- | --- | --- | --- | --- | --- |
| accessibility | fig N° | yellow  foundresses | black  foudresses | yellow  foundresses | black  foudresses | yellow  foundresses | black  foudresses |
| Whole day | 1 | 14 | 0 | 25 | 1 | 3 | 2 |
|  | 2 | 15 | 0 | 13 | 0 | 3 | 0 |
|  | 3 | 23 | 1 | 13 | 0 | 16 | 0 |
|  | 4 | 7 | 1 | 12 | 1 | 9 | 0 |
|  | 5 | 29 | 1 | 25 | 2 | 4 | 0 |
|  | 6 | 17 | 0 | 20 | 0 | 4 | 1 |
|  | 7 |  |  | 20 | 1 |  |  |
|  | 8 |  |  | 10 | 0 |  |  |
|  | 9 |  |  | 22 | 0 |  |  |
|  | 10 |  |  | 21 | 2 |  |  |
|  | 11 |  |  | 20 | 0 |  |  |
|  | 12 |  |  | 4 | 0 |  |  |
|  | 13 |  |  | 11 | 2 |  |  |
| Afternoon | 14 | 2 | 0 | 1 | 2 | 1 | 9 |
|  | 15 | 2 | 1 | 1 | 1 | 2 | 4 |
|  | 16 | 3 | 1 | 2 | 0 | 0 | 6 |
|  | 17 | 1 | 1 | 2 | 0 | 2 | 7 |
|  | 18 | 0 | 1 | 2 | 0 | 1 | 0 |
|  | 19 | 0 | 1 | 4 | 0 | 4 | 5 |
|  | 20 | 1 | 0 | 3 | 0 | 2 | 0 |
|  | 21 | 1 | 0 | 2 | 0 | 1 | 0 |
|  | 22 |  |  | 1 | 0 | 1 | 4 |
|  | 23 |  |  | 2 | 0 | 0 | 5 |
|  | 24 |  |  | 1 | 0 | 2 | 1 |
|  | 25 |  |  | 3 | 0 |  |  |
|  | 26 |  |  | 2 | 1 |  |  |
|  | 27 |  |  | 1 | 1 |  |  |
| Mann-Whitney  tests^1^ | W | 0 | 27 | 0.5 | 72 | 3 | 52.5 |
|  | p-value | 0.0023 | 0.71 | 1.03*10^-5 | 0.3 | 0.0026 | 0.047 |

^1^Compare the mean number of foundresses found within receptive figs according to their period of accessibility to pollinators.
