# Supplementary material for: Daily Rhythm of Mutualistic Pollinator Activity and Scent Emission in Ficus septica: Ecological Differentiation between Co-Occurring Pollinators and Potential Consequences for Chemical Communication and Facilitation of Host Speciation
Source: PLoS One. 2014 Aug 8;9(8):e103581. doi: 10.1371/journal.pone.0103581 (PMC4126690; doi:10.1371/journal.pone.0103581)
Supplement: Table S3 — Mean relative composition of the scents emitted by figs and leaves of Ficus septica and Ficus nota at sunrise and at noon. Mean relative contribution of each VOC to the scent of each sample category, expressed as a mean percentage +/− standard deviation. (DOCX) [file pone.0103581.s003.docx]

Supplementary table S3: Mean relative composition of the scents emitted by figs and leaves of *Ficus septica* and Ficus nota at sunrise and at noon. Mean relative contribution of each VOC to the scent of each sample category, expressed as a mean percentage +/- standard deviation .

|  |  | *Ficus nota* | | | | | | | | | | | | *Ficus septica* | | | | | | | | | | | |
| --- | --- | --- | --- | --- | --- | --- | --- | --- | --- | --- | --- | --- | --- | --- | --- | --- | --- | --- | --- | --- | --- | --- | --- | --- | --- |
|  |  | Figs | | | | | | Leaves | | | | | | Figs | | | | | | Leaves | | | | | |
|  |  | Sunrise | | | Noon | | | Sunrise | | | Noon | | | Sunrise | | | Noon | | | Sunrise | | | Noon | | |
| VOC identification | RI^1^ | mean | sd^2^ | O^3^ | mean | sd^2^ | O^3^ | mean | sd^2^ | O^3^ | mean | sd^2^ | O^3^ | mean | sd^2^ | O^3^ | mean | sd^2^ | O^3^ | mean | sd^2^ | O^3^ | mean | sd^2^ | O^3^ |
|  |  |  |  |  |  |  |  |  |  |  |  |  |  |  |  |  |  |  |  |  |  |  |  |  |  |
| *Fatty-acid derivatives* |  | *38.19* | *37.35* | *5* | *9.01* | *12.06* | *5* | *36.32* | *44.80* | *3* | *3.44* | *7.51* | *2* | *43.70* | *5.86* | *5* | *3.88* | *5.63* | *4* | *39.40* | *37.21* | *4* | *0.45* | *0.67* | *2* |
| (Z)-3-hexen-1-ol | 854 | 13.87 | 15.83 | 4 | 0.92 | 1.27 | 3 | 32.28 | 39.02 | 3 | 3.44 | 7.51 | 2 | 42.33 | 8.09 | 5 | 3.58 | 5.80 | 4 | 39.40 | 37.21 | 4 | 0.45 | 0.67 | 2 |
| (Z)-3-hexenyl-acetate | 1003 | 0.36 | 0.81 | 1 | 0.00 | 0.00 | 0 | 4.03 | 7.61 | 2 | 0.00 | 0.00 | 0 | 1.37 | 2.84 | 2 | 0.29 | 0.66 | 1 | 0.00 | 0.00 | 0 | 0.00 | 0.00 | 0 |
| 3-ethyl-4-methyl-pentan-1-ol | 1021 | 23.96 | 26.82 | 4 | 8.09 | 12.54 | 4 | 0.00 | 0.00 | 0 | 0.00 | 0.00 | 0 | 0.00 | 0.00 | 0 | 0.00 | 0.00 | 0 | 0.00 | 0.00 | 0 | 0.00 | 0.00 | 0 |
|  |  |  |  |  |  |  |  |  |  |  |  |  |  |  |  |  |  |  |  |  |  |  |  |  |  |
| *Terpenoids* |  | *48.57* | *33.09* | *5* | *83.09* | *14.64* | *5* | *61.83* | *44.24* | *5* | *94.47* | *8.53* | *5* | *47.88* | *12.46* | *5* | *89.68* | *3.58* | *5* | *60.06* | *37.05* | *5* | *94.23* | *4.92* | *5* |
| *Monoterpenoids* |  | *21.61* | *23.25* | *5* | *72.64* | *16.80* | *5* | *22.13* | *22.97* | *4* | *42.69* | *29.36* | *5* | *27.94* | *19.79* | *5* | *77.61* | *22.81* | *5* | *8.22* | *11.27* | *3* | *39.29* | *26.77* | *4* |
| α-thujene | 923 | 0.13 | 0.28 | 1 | 0.91 | 0.70 | 4 | 0.00 | 0.00 | 0 | 0.00 | 0.00 | 0 | 0.00 | 0.00 | 0 | 0.03 | 0.07 | 1 | 0.00 | 0.00 | 0 | 0.00 | 0.00 | 0 |
| α-pinene | 928 | 1.79 | 1.42 | 4 | 3.93 | 1.32 | 5 | 4.39 | 6.97 | 2 | 0.06 | 0.14 | 1 | 2.41 | 2.68 | 4 | 0.66 | 0.82 | 4 | 0.22 | 0.50 | 1 | 0.20 | 0.46 | 1 |
| sabinene | 968 | 3.63 | 5.17 | 5 | 22.45 | 6.05 | 5 | 0.00 | 0.00 | 0 | 0.55 | 1.24 | 1 | 1.78 | 3.98 | 1 | 3.93 | 4.24 | 5 | 0.00 | 0.00 | 0 | 0.00 | 0.00 | 0 |
| myrcene | 988 | 0.00 | 0.00 | 0 | 1.04 | 0.99 | 4 | 0.00 | 0.00 | 0 | 0.00 | 0.00 | 0 | 0.00 | 0.00 | 0 | 0.60 | 0.46 | 4 | 0.00 | 0.00 | 0 | 0.00 | 0.00 | 0 |
| δ-2-carene | 1005 | 0.17 | 0.37 | 1 | 0.00 | 0.00 | 0 | 10.24 | 22.90 | 1 | 0.00 | 0.00 | 0 | 0.04 | 0.09 | 1 | 0.00 | 0.00 | 0 | 0.00 | 0.00 | 0 | 0.11 | 0.25 | 1 |
| α-terpinene | 1014 | 0.00 | 0.00 | 0 | 0.30 | 0.24 | 4 | 0.00 | 0.00 | 0 | 0.00 | 0.00 | 0 | 0.00 | 0.00 | 0 | 0.01 | 0.02 | 1 | 0.00 | 0.00 | 0 | 0.00 | 0.00 | 0 |
| limonene | 1025 | 8.84 | 19.48 | 2 | 0.65 | 0.91 | 2 | 0.00 | 0.00 | 0 | 6.39 | 14.29 | 1 | 9.25 | 14.35 | 2 | 0.58 | 0.78 | 3 | 4.39 | 9.81 | 1 | 13.35 | 22.33 | 2 |
| 1,8-cineole | 1030 | 1.70 | 2.07 | 3 | 8.74 | 3.57 | 5 | 0.00 | 0.00 | 0 | 0.00 | 0.00 | 0 | 2.22 | 4.96 | 1 | 0.85 | 1.69 | 2 | 0.00 | 0.00 | 0 | 0.00 | 0.00 | 0 |
| (Z)-β-ocimene | 1035 | 0.14 | 0.30 | 1 | 2.04 | 1.54 | 4 | 0.00 | 0.00 | 0 | 1.68 | 2.37 | 2 | 0.00 | 0.00 | 0 | 4.93 | 1.53 | 5 | 0.00 | 0.00 | 0 | 3.66 | 5.38 | 2 |
| (E)-β-ocine | 1044 | 4.73 | 10.57 | 1 | 30.65 | 18.17 | 5 | 5.88 | 11.81 | 2 | 31.86 | 24.01 | 5 | 6.95 | 8.26 | 3 | 61.49 | 22.38 | 5 | 0.06 | 0.14 | 1 | 17.61 | 17.16 | 3 |
| γ-terpinene | 1056 | 0.06 | 0.14 | 1 | 0.69 | 0.48 | 4 | 0.00 | 0.00 | 0 | 0.00 | 0.00 | 0 | 0.00 | 0.00 | 0 | 0.04 | 0.06 | 2 | 0.00 | 0.00 | 0 | 0.00 | 0.00 | 0 |
| terpinolene | 1084 | 0.09 | 0.20 | 1 | 0.26 | 0.19 | 4 | 1.61 | 3.59 | 1 | 0.95 | 1.51 | 3 | 0.76 | 1.69 | 2 | 0.04 | 0.05 | 3 | 1.29 | 2.88 | 1 | 0.80 | 1.11 | 2 |
| linalool | 1098 | 0.12 | 0.26 | 1 | 0.34 | 0.37 | 4 | 0.00 | 0.00 | 0 | 0.27 | 0.52 | 2 | 4.54 | 6.09 | 4 | 1.98 | 3.22 | 4 | 2.26 | 5.05 | 1 | 2.74 | 4.97 | 2 |
| allo-ocimene | 1129 | 0.12 | 0.27 | 1 | 0.38 | 0.37 | 4 | 0.01 | 0.03 | 1 | 0.66 | 1.02 | 2 | 0.00 | 0.00 | 0 | 1.78 | 1.33 | 5 | 0.00 | 0.00 | 0 | 0.82 | 0.94 | 3 |
|  |  | *Ficus nota* | | | | | | | | | | | | *Ficus septica* | | | | | | | | | | | |
|  |  | Figs | | | | | | Leaves | | | | | | Figs | | | | | | Leaves | | | | | |
|  |  | Sunrise | | | Noon | | | Sunrise | | | Noon | | | Sunrise | | | Noon | | | Sunrise | | | Noon | | |
| VOC identification | RI^1^ | mean | sd^2^ | O^3^ | mean | sd^2^ | O^3^ | mean | sd^2^ | O^3^ | mean | sd^2^ | O^3^ | mean | sd^2^ | O^3^ | mean | sd^2^ | O^3^ | mean | sd^2^ | O^3^ | mean | sd^2^ | O^3^ |
| neo-allo-ocimene | 1141 | 0.10 | 0.22 | 1 | 0.09 | 0.10 | 3 | 0.00 | 0.00 | 0 | 0.27 | 0.37 | 2 | 0.00 | 0.00 | 0 | 0.40 | 0.62 | 3 | 0.00 | 0.00 | 0 | 0.00 | 0.00 | 0 |
| oxygenated monoterpene 1 | 1165 | 0.00 | 0.00 | 0 | 0.00 | 0.00 | 0 | 0.00 | 0.00 | 0 | 0.00 | 0.00 | 0 | 0.00 | 0.00 | 0 | 0.08 | 0.09 | 3 | 0.00 | 0.00 | 0 | 0.00 | 0.00 | 0 |
| p-mentha-1,5-dien-8-ol | 1170 | 0.00 | 0.00 | 0 | 0.00 | 0.00 | 0 | 0.00 | 0.00 | 0 | 0.00 | 0.00 | 0 | 0.00 | 0.00 | 0 | 0.08 | 0.11 | 2 | 0.00 | 0.00 | 0 | 0.00 | 0.00 | 0 |
| α-terpineol | 1179 | 0.00 | 0.00 | 0 | 0.14 | 0.14 | 3 | 0.00 | 0.00 | 0 | 0.00 | 0.00 | 0 | 0.00 | 0.00 | 0 | 0.00 | 0.00 | 0 | 0.00 | 0.00 | 0 | 0.00 | 0.00 | 0 |
| oxygenated monoterpene 2 | 1182 | 0.00 | 0.00 | 0 | 0.03 | 0.06 | 1 | 0.00 | 0.00 | 0 | 0.00 | 0.00 | 0 | 0.00 | 0.00 | 0 | 0.13 | 0.27 | 2 | 0.00 | 0.00 | 0 | 0.00 | 0.00 | 0 |
|  |  |  |  |  |  |  |  |  |  |  |  |  |  |  |  |  |  |  |  |  |  |  |  |  |  |
| *Sesquiterpenoids* |  | *26.83* | *32.20* | *5* | *9.71* | *7.25* | *5* | *29.28* | *34.65* | *5* | *1.87* | *2.14* | *3* | *19.94* | *12.03* | *5* | *7.76* | *14.93* | *5* | *48.53* | *40.09* | *5* | *8.14* | *6.69* | *4* |
| δ-elemene | 1330 | 0.00 | 0.00 | 0 | 0.00 | 0.00 | 0 | 0.00 | 0.00 | 0 | 0.00 | 0.00 | 0 | 1.08 | 1.49 | 2 | 0.00 | 0.00 | 0 | 0.00 | 0.00 | 0 | 0.00 | 0.00 | 0 |
| sesquiterpene 1 | 1365 | 0.00 | 0.00 | 0 | 0.03 | 0.07 | 1 | 0.00 | 0.00 | 0 | 0.00 | 0.00 | 0 | 0.00 | 0.00 | 0 | 0.00 | 0.01 | 1 | 0.00 | 0.00 | 0 | 0.00 | 0.00 | 0 |
| α-ylangene | 1366 | 0.26 | 0.46 | 2 | 0.21 | 0.26 | 4 | 4.51 | 10.09 | 1 | 0.00 | 0.00 | 0 | 0.00 | 0.00 | 0 | 0.01 | 0.01 | 1 | 0.00 | 0.00 | 0 | 0.03 | 0.06 | 1 |
| α-copaene | 1372 | 1.05 | 0.97 | 4 | 0.77 | 0.73 | 5 | 21.48 | 33.04 | 3 | 1.47 | 2.16 | 3 | 5.43 | 4.70 | 4 | 1.75 | 3.48 | 5 | 2.37 | 3.75 | 2 | 1.25 | 1.43 | 3 |
| 7-episesquithujene | 1386 | 0.40 | 0.62 | 3 | 0.13 | 0.17 | 3 | 0.00 | 0.00 | 0 | 0.00 | 0.00 | 0 | 0.00 | 0.00 | 0 | 0.00 | 0.00 | 0 | 0.00 | 0.00 | 0 | 0.00 | 0.00 | 0 |
| β-elemene | 1386 | 0.00 | 0.00 | 0 | 0.02 | 0.02 | 2 | 0.00 | 0.00 | 0 | 0.00 | 0.00 | 0 | 0.24 | 0.55 | 1 | 0.17 | 0.25 | 3 | 0.00 | 0.00 | 0 | 0.00 | 0.00 | 0 |
| sesquithujene | 1401 | 4.83 | 5.00 | 5 | 1.53 | 1.29 | 5 | 0.00 | 0.00 | 0 | 0.00 | 0.00 | 0 | 0.00 | 0.00 | 0 | 0.00 | 0.00 | 0 | 0.00 | 0.00 | 0 | 0.00 | 0.00 | 0 |
| cis-α-bergamotene | 1411 | 3.68 | 5.07 | 4 | 0.93 | 1.01 | 4 | 0.00 | 0.00 | 0 | 0.00 | 0.00 | 0 | 0.47 | 0.70 | 2 | 0.14 | 0.24 | 3 | 0.56 | 1.25 | 1 | 0.00 | 0.00 | 0 |
| sesquiterpene 2 | 1413 | 0.00 | 0.00 | 0 | 0.00 | 0.00 | 0 | 0.00 | 0.00 | 0 | 0.00 | 0.00 | 0 | 0.31 | 0.69 | 1 | 0.02 | 0.03 | 2 | 0.56 | 1.25 | 1 | 0.00 | 0.00 | 0 |
| (E)-caryophyllene | 1415 | 0.16 | 0.22 | 2 | 0.99 | 1.15 | 4 | 3.29 | 4.27 | 3 | 0.36 | 0.51 | 2 | 7.34 | 7.97 | 4 | 3.73 | 7.37 | 5 | 29.08 | 41.61 | 4 | 6.07 | 5.19 | 4 |
| α-santalene | 1417 | 7.95 | 9.54 | 5 | 2.21 | 1.73 | 5 | 0.00 | 0.00 | 0 | 0.00 | 0.00 | 0 | 0.00 | 0.00 | 0 | 0.00 | 0.00 | 0 | 0.00 | 0.00 | 0 | 0.00 | 0.00 | 0 |
| trans-α-bergamotene | 1430 | 5.35 | 7.50 | 4 | 1.40 | 1.49 | 4 | 0.00 | 0.00 | 0 | 0.00 | 0.00 | 0 | 0.29 | 0.43 | 2 | 0.24 | 0.50 | 3 | 0.00 | 0.00 | 0 | 0.00 | 0.00 | 0 |
| sesquiterpene 3 (selinene family) | 1443 | 0.88 | 1.41 | 2 | 0.37 | 0.39 | 4 | 0.00 | 0.00 | 0 | 0.00 | 0.00 | 0 | 0.00 | 0.00 | 0 | 0.00 | 0.00 | 0 | 0.00 | 0.00 | 0 | 0.00 | 0.00 | 0 |
| epi-β-santalene | 1445 | 0.21 | 0.35 | 2 | 0.15 | 0.20 | 3 | 0.00 | 0.00 | 0 | 0.00 | 0.00 | 0 | 0.00 | 0.00 | 0 | 0.00 | 0.00 | 0 | 0.00 | 0.00 | 0 | 0.00 | 0.00 | 0 |
| α-humulene | 1450 | 0.00 | 0.00 | 0 | 0.01 | 0.02 | 1 | 0.00 | 0.00 | 0 | 0.00 | 0.00 | 0 | 0.92 | 1.03 | 3 | 0.45 | 0.82 | 5 | 1.00 | 2.25 | 1 | 0.71 | 1.00 | 2 |
| (E)-β-farnesene | 1453 | 0.80 | 1.06 | 3 | 0.55 | 0.71 | 3 | 0.00 | 0.00 | 0 | 0.00 | 0.00 | 0 | 0.00 | 0.00 | 0 | 0.00 | 0.00 | 0 | 0.00 | 0.00 | 0 | 0.00 | 0.00 | 0 |
| allo-aromadendrene | 1455 | 0.00 | 0.00 | 0 | 0.00 | 0.00 | 0 | 0.00 | 0.00 | 0 | 0.04 | 0.08 | 1 | 1.22 | 2.30 | 2 | 0.02 | 0.03 | 2 | 0.00 | 0.00 | 0 | 0.00 | 0.00 | 0 |
|  |  | *Ficus nota* | | | | | | | | | | | | *Ficus septica* | | | | | | | | | | | |
|  |  | Figs | | | | | | Leaves | | | | | | Figs | | | | | | Leaves | | | | | |
|  |  | Sunrise | | | Noon | | | Sunrise | | | Noon | | | Sunrise | | | Noon | | | Sunrise | | | Noon | | |
| VOC identification | RI^1^ | mean | sd^2^ | O^3^ | mean | sd^2^ | O^3^ | mean | sd^2^ | O^3^ | mean | sd^2^ | O^3^ | mean | sd^2^ | O^3^ | mean | sd^2^ | O^3^ | mean | sd^2^ | O^3^ | mean | sd^2^ | O^3^ |
| sesquisabinene | 1457 | 0.04 | 0.08 | 1 | 0.07 | 0.16 | 1 | 0.00 | 0.00 | 0 | 0.00 | 0.00 | 0 | 0.00 | 0.00 | 0 | 0.00 | 0.00 | 0 | 0.00 | 0.00 | 0 | 0.00 | 0.00 | 0 |
| sesquiterpene 4 (cadinene family) | 1461 | 0.00 | 0.00 | 0 | 0.00 | 0.00 | 0 | 0.00 | 0.00 | 0 | 0.00 | 0.00 | 0 | 1.96 | 4.39 | 1 | 0.06 | 0.08 | 3 | 12.36 | 27.64 | 1 | 0.00 | 0.00 | 0 |
| sesquiterpene 5 (selinene family) | 1471 | 0.00 | 0.00 | 0 | 0.00 | 0.00 | 0 | 0.00 | 0.00 | 0 | 0.00 | 0.00 | 0 | 0.00 | 0.00 | 0 | 0.27 | 0.55 | 3 | 0.00 | 0.00 | 0 | 0.08 | 0.17 | 1 |
| ar-curcumene | 1481 | 0.04 | 0.09 | 2 | 0.05 | 0.12 | 1 | 0.00 | 0.00 | 0 | 0.00 | 0.00 | 0 | 0.00 | 0.00 | 0 | 0.44 | 0.95 | 2 | 0.00 | 0.00 | 0 | 0.00 | 0.00 | 0 |
| farnesene isomer | 1482 | 1.18 | 1.46 | 3 | 0.30 | 0.36 | 3 | 0.00 | 0.00 | 0 | 0.00 | 0.00 | 0 | 0.00 | 0.00 | 0 | 0.00 | 0.01 | 1 | 0.00 | 0.00 | 0 | 0.00 | 0.00 | 0 |
| bicyclogermacrene | 1492 | 0.00 | 0.00 | 0 | 0.00 | 0.00 | 0 | 0.00 | 0.00 | 0 | 0.00 | 0.00 | 0 | 0.37 | 0.84 | 1 | 0.00 | 0.00 | 0 | 2.61 | 5.83 | 1 | 0.00 | 0.00 | 0 |
| (E,E)-α-farnesene | 1503 | 0.00 | 0.00 | 0 | 0.00 | 0.00 | 0 | 0.00 | 0.00 | 0 | 0.00 | 0.00 | 0 | 0.00 | 0.00 | 0 | 0.06 | 0.09 | 2 | 0.00 | 0.00 | 0 | 0.00 | 0.00 | 0 |
| 7-epi-α-selinene | 1514 | 0.00 | 0.00 | 0 | 0.00 | 0.00 | 0 | 0.00 | 0.00 | 0 | 0.00 | 0.00 | 0 | 0.03 | 0.08 | 1 | 0.11 | 0.21 | 2 | 0.00 | 0.00 | 0 | 0.00 | 0.00 | 0 |
| δ-cadinene | 1516 | 0.00 | 0.00 | 0 | 0.00 | 0.00 | 0 | 0.00 | 0.00 | 0 | 0.00 | 0.00 | 0 | 0.27 | 0.61 | 1 | 0.28 | 0.63 | 2 | 0.00 | 0.00 | 0 | 0.00 | 0.00 | 0 |
|  |  |  |  |  |  |  |  |  |  |  |  |  |  |  |  |  |  |  |  |  |  |  |  |  |  |
| *Irregular terpenes* |  | *0.14* | *0.30* | *1* | *0.75* | *1.07* | *4* | *10.42* | *21.16* | *2* | *49.91* | *36.14* | *5* | *0.00* | *0.00* | *0* | *4.31* | *5.36* | *5* | *3.31* | *7.41* | *1* | *46.80* | *34.77* | *5* |
| (Z)-DMNT | 1092 | 0.00 | 0.00 | 0 | 0.00 | 0.00 | 0 | 0.00 | 0.00 | 0 | 1.00 | 1.82 | 2 | 0.00 | 0.00 | 0 | 0.19 | 0.23 | 3 | 0.00 | 0.00 | 0 | 6.71 | 6.22 | 4 |
| (E)-DMNT | 1111 | 0.14 | 0.30 | 1 | 0.75 | 1.07 | 4 | 10.42 | 21.16 | 2 | 48.91 | 36.09 | 5 | 0.00 | 0.00 | 0 | 4.12 | 5.41 | 5 | 3.31 | 7.41 | 1 | 40.09 | 36.00 | 5 |
|  |  |  |  |  |  |  |  |  |  |  |  |  |  |  |  |  |  |  |  |  |  |  |  |  |  |
| *Phenylpropanoids/benzenoids* |  | *10.56* | *17.57* | *5* | *5.37* | *3.26* | *5* | *1.86* | *3.50* | *2* | *2.08* | *2.03* | *3* | *8.42* | *16.08* | *3* | *6.30* | *3.45* | *5* | *0.54* | *1.20* | *1* | *5.32* | *5.00* | *4* |
| aromatic 1 | 1004 | 0.12 | 0.27 | 1 | 1.38 | 0.88 | 4 | 0.00 | 0.00 | 0 | 0.65 | 0.89 | 2 | 0.04 | 0.09 | 1 | 1.96 | 1.49 | 4 | 0.00 | 0.00 | 0 | 2.32 | 3.59 | 2 |
| p-cymene | 1022 | 0.05 | 0.11 | 1 | 0.98 | 0.83 | 4 | 0.00 | 0.00 | 0 | 0.00 | 0.00 | 0 | 0.00 | 0.00 | 0 | 0.00 | 0.00 | 0 | 0.00 | 0.00 | 0 | 0.00 | 0.00 | 0 |
| benzyl_alcohol | 1036 | 0.24 | 0.53 | 1 | 0.00 | 0.00 | 0 | 0.00 | 0.00 | 0 | 0.00 | 0.00 | 0 | 1.36 | 3.04 | 1 | 0.00 | 0.00 | 0 | 0.00 | 0.00 | 0 | 0.00 | 0.00 | 0 |
| aromatic 2 | 1078 | 0.00 | 0.00 | 0 | 0.34 | 0.27 | 4 | 0.00 | 0.00 | 0 | 0.00 | 0.00 | 0 | 0.00 | 0.00 | 0 | 0.33 | 0.35 | 3 | 0.00 | 0.00 | 0 | 1.55 | 3.47 | 1 |
| o-guaiacol | 1085 | 8.11 | 18.14 | 1 | 0.00 | 0.00 | 0 | 1.80 | 3.51 | 2 | 0.70 | 1.56 | 1 | 0.20 | 0.39 | 2 | 0.00 | 0.00 | 0 | 0.00 | 0.00 | 0 | 0.00 | 0.00 | 0 |
| p-cymenene | 1090 | 0.00 | 0.00 | 0 | 0.22 | 0.23 | 3 | 0.00 | 0.00 | 0 | 0.00 | 0.00 | 0 | 0.00 | 0.00 | 0 | 0.17 | 0.14 | 4 | 0.00 | 0.00 | 0 | 0.32 | 0.72 | 1 |
| aromatic 3 | 1092 | 0.00 | 0.00 | 0 | 0.09 | 0.13 | 2 | 0.00 | 0.00 | 0 | 0.00 | 0.00 | 0 | 0.00 | 0.00 | 0 | 0.07 | 0.14 | 2 | 0.00 | 0.00 | 0 | 0.00 | 0.00 | 0 |
| phenyl-ethyl-alcool | 1111 | 0.58 | 1.29 | 1 | 0.00 | 0.00 | 0 | 0.00 | 0.00 | 0 | 0.00 | 0.00 | 0 | 6.18 | 11.90 | 2 | 0.07 | 0.11 | 2 | 0.00 | 0.00 | 0 | 0.00 | 0.00 | 0 |
| aromatic 4 | 1120 | 0.16 | 0.35 | 1 | 0.43 | 0.46 | 3 | 0.04 | 0.09 | 1 | 0.08 | 0.17 | 1 | 0.00 | 0.00 | 0 | 0.67 | 0.44 | 4 | 0.00 | 0.00 | 0 | 0.30 | 0.68 | 1 |
|  |  | *Ficus nota* | | | | | | | | | | | | *Ficus septica* | | | | | | | | | | | |
|  |  | Figs | | | | | | Leaves | | | | | | Figs | | | | | | Leaves | | | | | |
|  |  | Sunrise | | | Noon | | | Sunrise | | | Noon | | | Sunrise | | | Noon | | | Sunrise | | | Noon | | |
| VOC identification | RI^1^ | mean | sd^2^ | O^3^ | mean | sd^2^ | O^3^ | mean | sd^2^ | O^3^ | mean | sd^2^ | O^3^ | mean | sd^2^ | O^3^ | mean | sd^2^ | O^3^ | mean | sd^2^ | O^3^ | mean | sd^2^ | O^3^ |
| aromatic 5 | 1130 | 0.30 | 0.46 | 2 | 1.57 | 1.56 | 4 | 0.01 | 0.03 | 1 | 0.66 | 1.02 | 2 | 0.00 | 0.00 | 0 | 3.04 | 1.54 | 5 | 0.00 | 0.00 | 0 | 0.82 | 0.94 | 3 |
| nitrogenated aromatic | 1164 | 0.51 | 0.86 | 2 | 0.11 | 0.24 | 2 | 0.00 | 0.00 | 0 | 0.00 | 0.00 | 0 | 0.00 | 0.00 | 0 | 0.00 | 0.00 | 0 | 0.00 | 0.00 | 0 | 0.00 | 0.00 | 0 |
| 4-ethenyl-1,2-dimethoxybenzene | 1363 | 0.00 | 0.00 | 0 | 0.00 | 0.00 | 0 | 0.00 | 0.00 | 0 | 0.00 | 0.00 | 0 | 0.60 | 1.19 | 2 | 0.00 | 0.00 | 0 | 0.00 | 0.00 | 0 | 0.00 | 0.00 | 0 |
| aromatic 6 | 1424 | 0.49 | 0.77 | 2 | 0.24 | 0.26 | 3 | 0.00 | 0.00 | 0 | 0.00 | 0.00 | 0 | 0.00 | 0.00 | 0 | 0.00 | 0.00 | 0 | 0.00 | 0.00 | 0 | 0.00 | 0.00 | 0 |
| aromatic 7 | 1517 | 0.00 | 0.00 | 0 | 0.00 | 0.00 | 0 | 0.00 | 0.00 | 0 | 0.00 | 0.00 | 0 | 0.03 | 0.08 | 1 | 0.00 | 0.00 | 0 | 0.54 | 1.20 | 1 | 0.00 | 0.00 | 0 |
| *Unidentified* |  | *2.68* | *2.51* | *4* | *2.53* | *5.07* | *4* | *0.00* | *0.00* | *0* | *0.00* | *0.00* | *0* | *0.00* | *0.00* | *0* | *0.14* | *0.11* | *4* | *0.00* | *0.00* | *0* | *0.00* | *0.00* | *0* |
| unidentified 1 | 943 | 1.94 | 2.69 | 4 | 2.40 | 5.14 | 3 | 0.00 | 0.00 | 0 | 0.00 | 0.00 | 0 | 0.00 | 0.00 | 0 | 0.00 | 0.00 | 0 | 0.00 | 0.00 | 0 | 0.00 | 0.00 | 0 |
| unidentified methyl-ester | 1050 | 0.58 | 0.83 | 2 | 0.00 | 0.00 | 0 | 0.00 | 0.00 | 0 | 0.00 | 0.00 | 0 | 0.00 | 0.00 | 0 | 0.00 | 0.00 | 0 | 0.00 | 0.00 | 0 | 0.00 | 0.00 | 0 |
| unidentified alcohol | 1086 | 0.00 | 0.00 | 0 | 0.12 | 0.11 | 3 | 0.00 | 0.00 | 0 | 0.00 | 0.00 | 0 | 0.00 | 0.00 | 0 | 0.09 | 0.06 | 4 | 0.00 | 0.00 | 0 | 0.00 | 0.00 | 0 |
| unidentified 3 | 1094 | 0.00 | 0.00 | 0 | 0.00 | 0.00 | 0 | 0.00 | 0.00 | 0 | 0.00 | 0.00 | 0 | 0.00 | 0.00 | 0 | 0.04 | 0.08 | 2 | 0.00 | 0.00 | 0 | 0.00 | 0.00 | 0 |
| unidentified 6 | 1227 | 0.12 | 0.26 | 1 | 0.01 | 0.02 | 1 | 0.00 | 0.00 | 0 | 0.00 | 0.00 | 0 | 0.00 | 0.00 | 0 | 0.00 | 0.00 | 0 | 0.00 | 0.00 | 0 | 0.00 | 0.00 | 0 |
| unidentified ketone | 1246 | 0.04 | 0.09 | 1 | 0.00 | 0.00 | 0 | 0.00 | 0.00 | 0 | 0.00 | 0.00 | 0 | 0.00 | 0.00 | 0 | 0.01 | 0.02 | 1 | 0.00 | 0.00 | 0 | 0.00 | 0.00 | 0 |
|  |  |  |  |  |  |  |  |  |  |  |  |  |  |  |  |  |  |  |  |  |  |  |  |  |  |
| total number of VOC emitted |  | 44 |  |  | 48 |  |  | 14 |  |  | 19 |  |  | 29 |  |  | 48 |  |  | 15 |  |  | 21 |  |  |

^1^Kovats retention index

^2^standard deviation

^3^occurrence = number of samples where the compound is found (n=5 for each sample category)
