# Supplementary material for: Daily Rhythm of Mutualistic Pollinator Activity and Scent Emission in Ficus septica: Ecological Differentiation between Co-Occurring Pollinators and Potential Consequences for Chemical Communication and Facilitation of Host Speciation
Source: PLoS One. 2014 Aug 8;9(8):e103581. doi: 10.1371/journal.pone.0103581 (PMC4126690; doi:10.1371/journal.pone.0103581)
Supplement: Table S4 — Main VOC responsible for additional differences of interest. P-values for the Mann-Whitney tests comparing the mean relative contribution of individual VOC between categories indicated by column titles. Each comparison is between groups of 10 samples. Significance codes (values non corrected for multiple testing): ns = non significant, *<0.05, **<0.01, ***<0.001. (DOCX) [file pone.0103581.s004.docx]

Table S4: Main VOC responsible for additional significant differences. P-values for the Mann-Whitney tests comparing the mean relative contribution of individual VOC to couples of sample categories as indicated by column titles. Each comparison is between groups of 10 samples. Significance codes (values non corrected for multiple testing): ns= non significant, *<0.05, **<0.01, ***<0.001

|  | Species difference  in figs | Fig-leaf difference  in *Ficus nota* | Fig-leaf difference in *Ficus septica* |
| --- | --- | --- | --- |
| *monoterpenes* |  |  |  |
| α-thujene | * | * | ns |
| α-pinene | ns | Ns | * |
| sabinene | * | *** | ** |
| myrcene | ns | * | * |
| α-terpinene | ns | * | ns |
| 1,8-cineole | * | *** | ns |
| (E)-β-ocimene | ns | Ns | * |
| γ-terpinene | ns | * | ns |
| linalool | * | Ns | ns |
| *sesquiterpenes* |  |  |  |
| α-ylangene | * | Ns | ns |
| 7-episesquithujene | ** | ** | ns |
| β-elemene | ns | Ns | * |
| sesquithujenene | *** | *** | ns |
| cis-α-bergamotene | ns | *** | ns |
| α-santalene | *** | *** | ns |
| trans-α-bergamotene | * | *** | * |
| sesquiterpene 3 | ** | ** | ns |
| epi-β-santalene | * | * | ns |
| α-humulene | ** | Ns | ns |
| allo-aromadendrene | * | Ns | * |
| (E)-β-farnesene | ** | ** | ns |
| sesquiterpene 4 | * | Ns | ns |
| farnesene isomer | * | ** | ns |
| *irregular terpenes* |  |  |  |
| (E)-DMNT | ns | * | ns |
| *phenylpropanoids-benzenoids* |  |  |  |
| p-cymene | * | * | ns |
| aromatic 2 | ns | * | ns |
| phenyl-ethyl-alcool | ns | Ns | * |
| nitrogenated aromatic | * | * | ns |
| aromatic 6 | * | * | ns |
| *fatty-acid-derivatives* |  |  |  |
| 3-ethyl-4-methyl-pentan-1-ol | *** | ** | ns |
| *unidentified* |  |  |  |
| unidentified 1 | ** | ** | ns |
| unidentified alcohol | ns | Ns | * |
